# Indoor air pollutants and respiratory symptoms among residents of an informal urban settlement in Uganda: A cross-sectional study

**DOI:** 10.1371/journal.pone.0290170

**Published:** 2023-08-17

**Authors:** Solomon T. Wafula, Aisha Nalugya, Hilbert Mendoza, Winnifred K. Kansiime, Tonny Ssekamatte, Abel W. Walekhwa, Richard K. Mugambe, Florian Walter, John C. Ssempebwa, David Musoke

**Affiliations:** 1 Department of Disease Control and Environmental Health, School of Public Health, Makerere University, Kampala, Uganda; 2 Department of Infectious Disease Epidemiology, Bernhard Nocht Institute for Tropical Medicine, Hamburg, Germany; 3 Department of Family Medicine and Population Health, Faculty of Medicine and Health Sciences, University of Antwerp, Wilrijk, Belgium; 4 Division of Nursing, Midwifery and Social Work, School of Health Sciences, Faculty of Biology, Medicine and Health, University of Manchester, Manchester, United Kingdom; Universita del Piemonte Orientale, ITALY

## Abstract

**Background:**

Indoor air pollutants (IAP) and household conditions such as dampness, crowding and chemical exposures have been associated with acute and chronic respiratory infections. In Uganda, literature on the effects of IAP on respiratory outcomes in informal settlements is limited.

**Methods:**

We describe the baseline household characteristics of 284 adults and their children in an informal settlement in Uganda from April to May 2022. We monitored same-day indoor concentrations of particulate matter PM_2.5_, PM_10_, Carbon monoxide (CO), relative humidity %, and temperature from 9 am to 2 pm and interviewed caregivers/mothers about their respiratory symptoms and those of their children in the previous 30 days. We employed robust Poisson regressions to evaluate the associations between indoor air indicators and respiratory health symptoms.

**Results:**

Approximately 94.7% of households primarily used biomass fuels and 32.7% cooked from inside their dwelling rooms. The median PM_2.5_, PM_10_ and CO levels were 49.5 (Interquartile range (IQR) = 31.1,86.2) μg/m^3^, 73.6 (IQR = 47.3,130.5) μg/m^3^ and 7.70 (IQR = 4.1,12.5) ppm respectively. Among adults, a 10 unit increase in PM_2.5_ was associated with cough (Prevalence Ratio (PR) = 3.75, 95%CI 1.15–1.55). Dwelling unit dampness was associated with phlegm (PR = 2.53, 95%CI = 1.39–4.61) and shortness of breath (PR = 1.78, 95% CI 1.23–2.54) while cooking from outside the house was protective against shortness of breath (PR = 0.62, 95% CI = 0.44–0.87). In children, dampness was associated with phlegm (PR = 13.87, 95% CI 3.16–60.91) and cough (PR = 1.62, 95% CI 1.12–2.34) while indoor residual spraying was associated with phlegm (PR = 3.36, 95%CI 1.71–6.61).

**Conclusion:**

Poor indoor air conditions were associated with respiratory symptoms in adults and children. Efforts to address indoor air pollution should be made to protect adults and children from adverse health effects.

## Introduction

Indoor air pollution (IAP) is a major environmental and public health challenge in low–and middle-income countries (LMICs), including Uganda [1]. Approximately two million people die prematurely per year from illnesses attributable to IAP from solid fuels such as charcoal and firewood. IAP can be caused by using solid fuels for cooking, lighting candles or oil lamps, fuel-burning space heaters, smoking, and indoor residual insecticide spraying. In LMICs, inefficient and inadequately vented cooking spaces and heating with biomass and coal on simple stoves are critical [2, 3]. Burning these fuels in inefficient stoves produces high levels of health-damaging air pollutants such as particulate matter (PM), carbon monoxide (CO) and a variety of volatile organic compounds (VOCs) [4].

The quantities emitted and relative composition of different emissions are determined by various factors, including the type of fuel, humidity, dampness, stove type and the way the stove and fuel are used by the cook [5]. There is growing evidence that poor IAP and poor housing conditions can lead to various respiratory health problems, such as coughing, wheezing, shortness of breath, chest tightness, irritation of the eyes, nose, and throat, and asthma which can have a significant impact on individuals’ quality of life [6–8]. Additionally, long-term health effects such as pneumonia, chronic obstructive pulmonary disease, heart disease, and lung cancer have also been documented to be associated with exposure to IAP [9]. Evidence suggests that women and children have a higher risk of adverse health effects than men due to prolonged exposure to biomass smoke during food preparation at home and spending much time indoors [10].

There are evident socioeconomic disparities in biomass fuel use, with economically poor households more likely to use unimproved fuels [11, 12]. Indeed, in Uganda, most urban poor live in informal settings such as slums. Informal settings are overcrowded, poorly ventilated spaces and structures are not constructed following existing building codes [13]. Inadequate housing conditions in Ugandan informal settings can negatively affect indoor air quality and hence partly account for disparities in the burden of ARIs [7]. Slum dwellers largely use biomass fuels and tend to cook indoors; conditions that result in elevated levels of air pollutants such as PM, CO, and other VOCs [14]. Our previous study set in an urban informal setting showed 24hr mean PM_2.5_ concentrations of 69.62 μg/m^3^ which are more than four times higher than 15 μg/m^3^, the World Health Organization (WHO) recommendations.

Studies in high-income countries have reported significant associations between respiratory infections and indoor sources of air pollutants, materials or activities, e.g., recent painting, VOCs, gas appliances, biomass fuel use and exposure to household smoking [15, 16]. However, limited studies especially in sub-Saharan Africa (SSA) have objectively measured concentrations of IAPs which would enable a more robust assessment of the associations with respiratory effects if equipment are calibrated and validated [17]. Instead, the studies in SSA depend on self-reports to assess exposures related to IAP [18], which is subject to inherent biases [19]. Although relying on sources of IAP may be a good proxy of exposure, objectively measured quantitative exposure assessments can be more helpful in assessing the health effects of exposure.

We aimed to investigate the association between selected indoor air pollutants, cooking fuels and objectively measured concentrations of air pollutants and respiratory symptoms among children and adults in an informal urban setting in Uganda. While there is a growing body of evidence that IAP is associated with respiratory health problems, there is a need for further research to better understand the relationship between indoor air quality and respiratory health using various non-specific respiratory symptoms. The use of non-specific respiratory symptoms can provide a more comprehensive understanding of the broader impact of indoor air quality on respiratory health. This is particularly important given the negative impact poor indoor air quality can have on an individual’s quality of life.

## Methods

### Study setting

**Fig 1:**
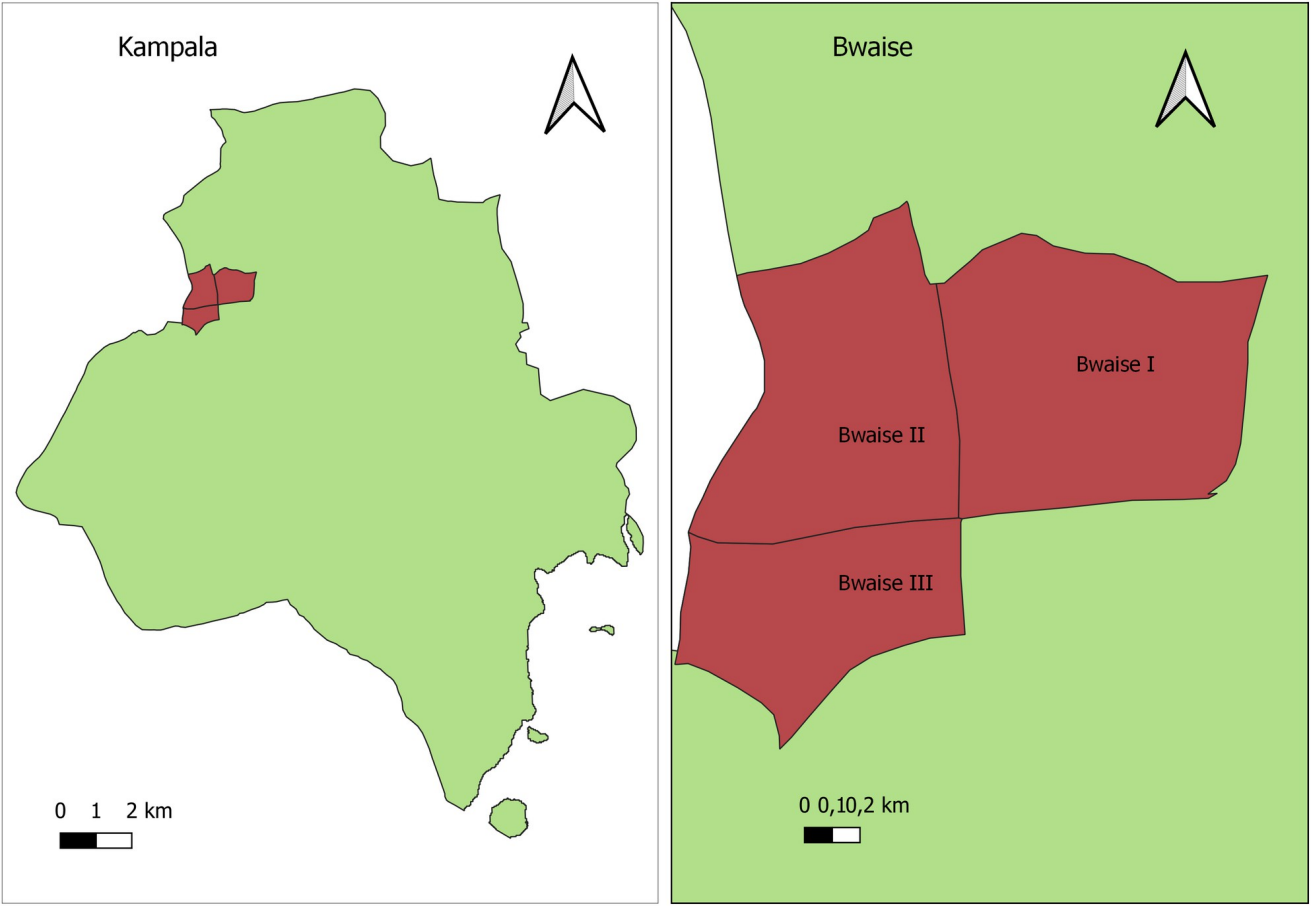
Map Showing the location of Bwaise within Kampala.

This study was conducted in Bwaise slum, an informal settlement in Kawempe Division, Kampala, Uganda (Fig 1). This informal settlement is one of Kampala’s most densely populated slums, distinguished by largely informal and substandard housing and small-scale businesses. It has a large population density, congested households with low socioeconomic status and high dependence on solid fuels [20]. Therefore, we expected higher pollution levels in such a slum than in other outdoor settings.

### Study design, area and population

This community-based cross-sectional survey used questionnaires and an observational checklist to collect data on self-reported respiratory symptoms, including coughing, wheezing, and phlegm, shortness of breath among mothers/caregivers (aged 18 years and above) and children (aged 0 to 5 years) in Bwaise 1, Bwaise II and Bwaise III slums of Kawempe Division, Kampala City Council Authority (KCCA), Uganda. Approximately 60% of the urban residents in Kampala city reside in informal areas [13]. The neighborhoods of Bwaise I, Bwaise II, and Bwaise III have an average population of approximately 37,500, 42,000, and 35,000 people, respectively [21]. These areas are characterized by cramped living conditions, with an average household size ranging from 5 to 10 individuals. Typically, these households occupy small dwellings consisting of just one or two rooms [22]. Information on household and individual risk factors was collected from participants from April to May 2022.

The eligible study population consisted of adults (18 years and above) from households who had resided in the area for at least three months, not incapacitated, and would provide informed consent. Those who had severe mental illnesses or declined consent were excluded. Participants provided information on the demographics and respiratory health within the previous 30 days of all children (0–59 months) under their care/household.

### Sample size and sampling procedure

Using the Kish Leslie formulae for cross-sectional studies [23], we intended to sample 278 households using a prevalence of self-reported health effects (respiratory symptoms) of 19.4% from a previous study in Uganda, considering a 5% level of significance, 80% power and non-response rate of 15%.

As regards sampling, Bwaise slum was selected purposively because of poor housing and living conditions and over-reliance on solid/biomass fuels. Within each of the three Bwaise parishes, one zone was chosen randomly using the ballot method, and approximately 93 households were selected in each zone using systematic sampling. The sampling interval for each zone was obtained by dividing the number of households (according to records from the zonal local council office) by the number of households needed (i.e., divided by 93). In each zone, we started sampling at the zonal local council office, north direction, and clockwise until we obtained the sample. The following household replaced a selected household if the original household had no eligible respondents or did not consent. In each selected household, one adult or another person generally involved in the cooking responded to the questionnaire. We obtained information from adults on respiratory symptoms among all children under five years in their selected household.

### Data collection and measurements

We developed data collection tools and captured relevant covariates following a thorough review of the existing literature [24–27]. Six experienced and trained data collectors with background training in Environmental Health Science administered the tools to eligible participants and captured responses on Kobo Collect, a mobile data collection app [28].

■ Respiratory health: The study’s primary outcomes were self-reported respiratory symptoms among adults and children (reported by parents/guardians) within the last 30 days. These symptoms included cough, phlegm, wheezing, shortness of breath and blocked/running nose and these were responses to the multiple-choice question “Have you had the following symptoms in the last 30 days on at least three days?”. For children, the question was adapted to read “Has ‘(name of the child)’ had the following symptoms in the past 30 days for at least 3 days?” and the responses were yes (coded 1) or no (coded 0)
■ Indoor air conditions: Information was collected on parameters such as indoor dampness, presence of indoor plants (yes or no), and indoor residual spraying (yes or no, and on resident behaviours including smoking (yes or no), biomass fuel use (yes or no), carpet use (yes or no), and cooking place location (indoor kitchen vs outdoors). We defined dampness as evidence of signs of mould growth, moisture stains and peeling walls in indoor spaces [29]. We also asked participants about their use of indoor residual spraying (IRS) since it is one of the vector control methods for malaria given its endemicity in Uganda. This was self-reported. We measured indoor particulate matter of 2.5 microns (PM_2.5_), particulate matter of 10 microns (PM_10_), carbon monoxide (CO), humidity (RH)%, and temperature (°Con the same day ten times at each household over the course of five hours (twice per hour from 9 am to 2 pm) to reduce variability than a single measurement. For each of the ten sampling events, the three-minute average concentrations (mean of three readings per event) of PM_2.5_, PM_10_, CO, humidity, and temperature were measured in the centre of the living room. All meters were placed on the table/platform one meter above the ground. Due to reduced airflow near surfaces, the monitors’ air receivers and inlets were placed at least 1.5 m away from the windows and doors [30]. Three low-cost particle sensors (Temptop M2000c 2^nd^ edition) were used to measure particulate matter. Temtop M2000c 2^nd^ sensor utilizes an optical particle counter and its detectors have a laser particle sensor and an operating temperature range of 0–50°C, a relative humidity range of 0–90%, an atmospheric pressure of one atm, and a PM_2.5_ measurement range of 0–999 ug/m3 with a resolution of 0.1 ug/m3. Temptop monitors were factory calibrated, and therefore further laboratory calibration was not required for the duration of the study. CO levels were also measured using three AS8700A CO meter, which has a detection range of 0~1000 ppm and with accuracy (± 5% or ± 10 ppm). The temperature was expressed in degrees Celsius (°C), and humidity was shown as a percentage.
■ Covariates: We collected data covariates and potential confounding variables including socio-demographic characteristics such as gender (male or female), age in complete years (adults) and in months (children), marital status (single, married, separated), occupation (employed, unemployed, others), monthly household income and length of residence (in years). The length of the stay was categorized into three: (i) less than or equal to 5 years, (ii) 6 to 10 years, and (iii) more than 10 years. Household income in Uganda shillings to US Dollars (I USD = 3500 UGX) and categorized as follows: (i) less than 50 USD, (ii) between 50 to 150 USD, and (iii) greater than 150 USD. We used the median national monthly household income of around 50 USD as the cut-off for the first category, while the second category upper limit was based on the median monthly income of approximately 150 USD for the capital, Kampala) [13]. Tools were pretested among 20 mothers/caregivers in the Katanga slum, Kampala, which has similar characteristics to Bwaise. Details on the definitions of covariates and simple directed acyclic graph (DAG) is provided in **S1 Text**.

### Statistical analysis

We used the median and interquartile range (IQR) to describe continuous variables, and frequencies and percentages to describe categorical variables. For the associations between indoor air conditions and selected respiratory symptoms among caregivers/mothers and children, we performed separate multivariable modified Poisson regressions [31], producing prevalence ratios (PRs) and corresponding 95% confidence interval (95%CI) adjusting for age, gender, smoking status. The outcomes-: respiratory symptoms cough, phlegm, wheezing, and shortness of breath. For this model, we included two latent variables, each calculated as one-tenth of the logarithmically transformed average concentrations of the original PM2.5 and PM10 values, respectively. To account for potential confounding factors, we included additional covariates in the multivariable model, along with the well-established confounders such as age, gender, and smoking. These additional covariates were selected based on a priori knowledge and guided by the Directed Acyclic Graph (DAG) to ensure comprehensive adjustment for potential confounding effects. The data were analyzed using the statistical software Stata 14.0 version. All statistical tests were two-tailed, and statistical significance was assumed when a p value was < = 0.05.

### Ethical consideration

We obtained the study’s ethical approval from Makerere University School of Public Health Research and Ethics Committee (Ref No. SPH-2021-99) and the Uganda National Council of Science and Technology (UNCST: Ref No. SS996ES). Administrative clearance was sought from Kampala City Council Authority (KCCA), which presides over the study area. Information sheets and consent forms were available in the local language (Luganda) or English with details on the purpose of the project, procedures to be followed and the risks and benefits of participation. We obtained written informed consent from each participant (adults). We assured participants of the confidentiality and anonymity of their information. We advised participants in households with higher particulate matter levels to adopt appropriate exposure mitigation strategies, and we advised those with chronic respiratory infections to seek medical attention.

## Results

### Background characteristics of the participants

Out of the 284 adult participants who took part in the study, the majority (85.2%) were females and 51.4% of the participants were below the age of 30 years m. The median age (IQR) was 29 years. Nearly half, 47.9%, were married or living with a partner, 51.1% had attained a post-primary level of education, and 68.6% were employed. More than half, 57.4%, had a monthly household income between 50–150 US dollars, and 52.1% had stayed in their current area of residence for less than five years. About 14.4% (41) adults had smoked in the previous 30 days. Of the 284 households, 187 had at least a child below the age of 5. The median age of the children was 24.0 months (IQR = 4,48). Of these, 52.6% were females, and 72.2% were not in school. Information was also obtained on 230 children under 5 (52.6% Female; 72.2% were not yet in school) (Table 1).

**Table 1:** Background characteristics of adult household participants.

Characteristics	Number of participants,	Percentage
	*N* = 284	(%)
**Gender:** Female	242	85.2
**Age of respondents (in years)**		
< 30	146	51.4
30–45	108	38.0
> 45	30	10.6
**Marital status**		
Married or living with a partner	136	47.9
Separated	43	15.1
Single	105	37.0
**Education level**		
No formal education	25	8.8
Primary	114	40.1
Post-primary	145	51.1
**Occupation**		
Employed	195	68.6
Unemployed	72	25.4
Other	17	6.0
**Owner of the dwelling**	26	9.2
**Household monthly income (USD)**		
< 50	77	27.1
50–150	163	57.4
> 150	44	15.5
**Duration of stay in the area of residence (years)**		
< 5	148	52.1
5–10	57	20.1
> 10	79	27.8
Current smoker		
No	243	85.6
Yes	41	14.4

### Cooking fuels, indoor conditions and air pollutant levels

Of 284 households, 269 (94.7%) used biomass fuels (wood and charcoal). The median PM_2.5_, PM_10_ and CO levels were 49.5 (IQR = 31.1,86.2) μg/m^3^, 73.6 (IQR = 47.3,130.5) μg/m^3^ and 7.70 (4.1,12.5) ppm respectively. Dampness was found in 123 (43.3%) of the households (Table 2).

**Table 2:** Cooking fuels, indoor conditions and air pollutant levels.

Indoor air parameters	Number, N = 284	Summary statistic
**Meteorological parameters (**Median (IQR))		
Humidity	272	70.2 (66.9, 73.2)
Temperature	272	28.2 (27.2, 29.1)
**Air quality parameters (**Median (IQR))		
PM _2.5_ (μg/m^3^)	272	49.5 (31.1–86.2)
PM_10_ (μg/m^3^)	272	73.6 (47.3,130.5)
Carbon monoxide (ppm)	272	7.70 (4.1,12.5)
**Main fuel type**		
Biomass	269	94.7%
Non-biomass	15	5.3%
**Cooking from outside**	194	68.3%
**Rearing pets**	25	8.8%
**Carpets in living room**	127	44.7%
**Home dampness (mould)**	123	43.3%

Note: For fuel type, cooking from outside, rearing pets, carpets in living room and dampness (mould); the summary statistic is percentages otherwise median and interquartile range

### Respiratory symptoms among adults and children

Of the 284 adults, 66.2% reported coughing, 41.9% reported a running nose, 33.5% reported shortness of breath, 17.6% reported phlegm and 14.8% reported wheezing. Most respondents (84.6%) reported having at least one of these respiratory symptoms in the previous 30 days. A total of 230 children were included in this study, of which 80.0% had morning cough, 44.8% had a running nose, 34.4% reported day or night cough, 26.5% had shortness of breath, 20.0% had wheezing, and 13.5% had phlegm during the previous 30 days. The distribution of respiratory outcomes among participants is presented in **S1** and **S2 Tables** for adults and children, respectively.

### Associations between indoor air conditions and respiratory problems among adults

At multivariable analysis, the respondents with dampness in their dwelling units reported a higher prevalence of phlegm (PR = 2.53, 95%CI = 1.39–4.61) and shortness of breath (PR = 1.78, 95% CI = 1.23–2.54). Similarly, respondents whose cooking place was located outside their living house reported a 38% lower risk of shortness of breath (PR = 0.62, 95% CI = 0.44–0.87) and 15% lower risk of cough (PR = 0.85, 95% CI = 0.71–0.99) than those whose cooking place was inside the living rooms. A 10 unit increase in PM_2.5_ levels was associated with increased risk of cough (PR = 3.75, 95%CI = 1.15–12.10) among adults. Use of indoor residual sprays was associated with shortness of breath (PR = 1.44, 95%CI = 1.02–2.03) while pet rearing was associated with cough (PR = 1.31, 95% CI = 1.11–1.55) (Table 3).

**Table 3:** Adjusted models for association between indoor air quality parameters and respiratory problems among adult residents.

Attributes	Cough	Phlegm	Wheezing	Blocked /Runny nose	Shortness of breath
	PR (95% CI)	PR (95% CI)	PR (95% CI)	PR (95% CI)	PR (95% CI)
Demographic characteristics					
Gender: Male	1.12 (0.89–1.40)	1.26 (0.64–2.47)	0.15 (0.02–1.15)	0.92 (0.56–1.52)	0.84 (0.53–1.32)
Age (in complete years)					
< 30	1	1	1	1	
30–45	1.02 (0.86–1.20)	0.94 (0.52–1.68)	1.44 (0.80–2.59)	0.95 (0.71–1.28)	0.94 (0.67–1.33)
45 +	0.75 (0.53–1.06)	0.88 (0.40–1.92)	0.89 (0.26–3.01)	0.42 (0.21–0.85)	0.78 (0.43–1.43)
Education level					
Non-formal	1	1	1	1	1
Primary	0.93 (0.71–1.20)	1.87 (0.58–6.01)	0.54 (0.23–1.27)	0.89 (0.47–1.67)	1.29 (0.66–2.52)
Post-primary	0.95 (0.74–1.23)	1.27 (0.38–4.12)	0.47 (0.20–1.12)	1.08 (0.59–1.98)	1.09 (0.55–2.18)
Occupation					
Employed (including business)	1	1	1	1	1
Unemployed	0.86 (0.69–1.07)	0.77 (0.38–1.54)	0.67 (0.29–1.54)	1.09 (0.78–1.52)	0.81 (0.54–1.21)
Other	0.79 (0.53–1.18)	0.59 (0.18–1.89)	1.40 (0.49–4.01)	1.93 (1.22–3.04)	0.28 (0.08–0.90)
Housing characteristics					
HH Income					
< 50	1	1	1	1	1
50–150	1.08 (0.88–1.33)	1.63 (0.82–3.21)	1.58 (0.69–3.63)	0.93 (0.65–1.32)	1.09 (0.73–1.63)
>150	1.08 (0.82–1.43)	0.73 (0.22–2.39)	1.29 (0.43–3.83)	1.56 (1.05–2.33)	0.95 (0.52–1.72)
Cooking outside living house	0.85 (0.71–0.99)	0.88 (0.49–1.55)	0.77 (0.42–1.39)	1.10 (0.82–1.49)	0.62 (0.44–0.87)
PM _2.5_^1^	3.75 (1.15–12.10)	1.21 (0.04–39.89)		1.14 (0.14–9.62)	2.27 (0.20–26.31)
Carbon monoxide	1.00 (0.99–1.01)	1.01 (0.98–1.02)	0.94 (0.91–1.07)	1.00 (0.99–1.01)	0.98 (0.97–1.00)
Main fuel: Biomass	1.75 (0.94–3.25)	1.83 (0.26–12.76)	1.24 (0.20–7.84)	0.94 (0.55–1.62)	0.94 (0.44–1.98)
Rearing pets	1.31 (1.11–1.55)	1.11 (0.53–2.30)	1.51 (0.69–3.31)	0.81 (0.47–1.39)	1.20 (0.72–2.03)
Carpets in the living room	0.92 (0.77–1.10)	0.91 (0.54–1.54)	0.59 (0.30–1.15)	0.93 (0.70–1.23)	0.88 (0.62–1.23)
Home dampness	0.99 (0.84–1.18)	2.53 (1.39–4.61)	1.46 (0.81–2.62)	0.89 (0.65–1.22)	1.78 (1.23–2.54)
Smoker	1.12 (0.93–1.35)	1.52 (0.79–2.93)	0.96 (0.38–2.40)	0.97 (0.62–1.52)	0.87 (0.54–1.41)
Indoor spraying for insecticides	1.15 (0.98–1.36)	1.21 (0.70–2.09)	1.09 (0.59–2.00)	0.95 (0.69–1.31)	1.44 (1.02–2.03)

Note: ^1^ PM2.5; 1/10 of log transformed PM2.5 average values; All models adjusted for age, gender, income, and education and smoking

### Associations between indoor air conditions and respiratory problems among parent-reported respiratory problems among children

At the multivariable level, the prevalence of phlegm among children in households that did indoor spraying of insecticides (PR = 3.36, 95% CI = 1.71–6.61) was 3.4 times higher compared to that among households that did not. Dampness was associated with Increased risk of phlegm (PR = 13.87, 95% CI 3.16–60.91) and day/night cough (PR = 1.62, 95% CI 1.12–2.34). Smoking by the parent/ guardian was associated with an increased risk of running nose while pet rearing was associated with wheezing (PR = 1.74, 95% CI 1.03–3.23) (Table 4).

**Table 4:** Associations between indoor air quality conditions and respiratory problems among children (adjusted analysis).

Attributes	Morning Cough	Day or night cough	Phlegm	Wheezing	Blocked / Runny nose	Shortness of breath
**Demographic characteristics**						
**Gender:** Male	1.06 (0.93–1.20)	0.66 (0.46–0.95)	0.98 (0.52–1.83)	0.88 (0.52–1.49)	0.81 (0.61–1.07)	0.85 (0.55–1.33)
**Age (in complete years)**						
**< = 2**	1	1	1	1	1	1
**2+**	1.06 (0.92–1.22)	1.32 (0.89–1.97)	1.05 (0.56–1.95)	1.36 (0.77–2.41)	1.32 (0.97–1.78)	1.11 (0.70–1.75)
**Child Education**						
Not in school	1	1	1	1	1	1
School	1.00 (0.86–1.15)	1.02 (0.69–1.52)	0.86 (0.43–1.72)	0.60 (0.29–1.23)	0.89 (0.64–1.24)	0.71 (0.39–1.28)
**Housing conditions**						
**Cooking place: Outside**	1.05 (0.90–1.23)	1.25 (0.84–1.85)	0.69 (0.38–1.25)	0.63 (0.36–1.10)	0.82 (0.61–1.10)	0.80 (0.51–1.24)
PM_2.5_^1^	1.23 (0.47–3.23)	0.45 (0.03–6.36)	6.06 (0.08–457.4)	1.98 (0.04–110.98)	0.22 (0.02–2.47)	0.88 (0.63–1.22)
Carbon monoxide	1.00 (0.99–1.00)	0.99 (0.97–1.01)	1.02 (1.00–1.04)	0.98 (0.95–1.01)	0.99 (0.98–1.01)	1.01 (0.99–1.02)
**Main fuel:** Biomass	0.88 (0.67–1.14)	1.46 (0.47–4.56)		1.56 (0.23–10.33)	1.18 (0.52–2.70)	1.15 (0.33–4.02)
**Rearing pets**	0.89 (0.70–1.14)	1.29 (0.78–2.12)	1.08 (0.50–2.32)	1.74 (1.03–3.23)	0.56 (0.32–1.00)	0.96 (0.47–1.97)
**Carpets in the living room**	1.06 (0.93–1.21)	1.31 (0.90–1.92)	1.08 (0.59–1.98)	1.13 (0.66–1.93)	0.90 (0.67–1.20)	1.15 (0.742–1.84)
**Home dampness**	1.09 (0.96–1.25)	1.62 (1.12–2.34)	13.87 (3.16–60.9)	1.53 (0.88–2.68)	1.26(0.95–1.67)	1.32 (0.83–2.11)
**Parent / guardian smokes**	0.94 (0.75–1.17)	0.65 (0.37–1.12)	0.60 (0.25–1.46)	0.95 (0.48–1.89)	1.46 (1.06–2.00)	0.87 (0.44–1.72)
**Indoor spraying for insecticides**	1.00 (0.48–1.16)	0.65 (0.44–1.00)	3.36 (1.71–6.61)	1.14 (0.67–1.46)	0.50 (0.33–1.17)	0.83 (0.52–1.34)

Note: ^1^ PM2.5; 1/10 of log transformed PM2.5 average values; All models adjusted for child’s age, gender, education and regular smoking by household member.

## Discussion

Understanding the impact of indoor air pollution on respiratory health is critical because many people spend nearly two-thirds of their time at home [14]. This is one of the first studies to investigate the association between indoor air quality conditions and respiratory symptoms among adults and children in informal settlements in Uganda. Higher than normal ranges of indoor PM_2.5_ and PM_10_ levels were observed in most study households. Among adults, increases in PM_2.5_ was associated with cough and wheezing. Dwelling unit dampness was associated with phlegm and shortness of breath while outside cooking was protective against cough and shortness of breath. In children, dampness was associated with phlegm and cough, pet rearing was associated with wheezing while indoor residual spraying was associated with phlegm.

We found median PM_2.5_ and PM_10_ levels of 49.6 μg/m^3^ and 73.6 μg/m^3^. Limited studies have measured indoor PM_2.5_ and PM_10_ concentrations in Uganda although Kansiime et al. [32] reported mean indoor PM_2.5_ concentrations of 124.29 μg/m^3^ in Fort Portal city based on single-time measurements. The high levels particulate matter levels highlights may imply increase in risk to human health including respiratory problems, cardiovascular diseases, and reduced life expectancy. Most households relied on biomass fuels which due to inefficient burning emit higher levels of pollutants, including particulate matter and CO [33]. We therefore suggest a need for interventions that promote clean fuels such as gas and electricity. The effect of fuel type on respiratory health could not be robustly determined since nearly all households used biomass fuels.

This study revealed a high prevalence (84.6%) of self-reported acute respiratory symptoms (at least one symptom) in adults, which included wheezing, cough and phlegm in the last 30 days before the survey. Likewise, 80% of children had morning cough, 44.8% reported a runny nose, 34.4% reported day or night cough, and 26.5% reported shortness of breath at least three times during the preceding 30 days. Although self-reported, the high prevalence of respiratory symptoms highlights the significant burden among residents and requires efforts to reduce associated risk factors. The prevalence of respiratory symptoms in our study population was much higher than the 20% prevalence which was assumed in the sample size calculation [34]. The high prevalence of respiratory symptoms in the study population may be attributed to the high levels of IAP in the study area and the widespread use of solid fuels for cooking. Moreover, our study was conducted in an informal settlement in Kampala, where indoor air quality conditions are significantly poorer than in upscale settings. In contrast, the study by siddharthan [34] was conducted in both rural and urban Uganda. This marked differences in setting could partly explain the high prevalence of respiratory symptoms in our study than the 20% assumed in the sample size calculation. The high prevalence underscores the urgent need for interventions to improve indoor air quality in slum settings.

Our study identified showed that elevated risk of cough with 10 unit increase in PM_2.5_ levels in adults. Although this is among the first studies to document this association in Uganda, the finding is consistent with published evidence elsewhere in high-income countries [35–37]. Indoor PM concentration is related to inflammation and a decrease in lung function [38]; hence cough can be one of the manifestations of this effect. This underscores the need to reduce all activities that may encourage the deposition of particulate matter in indoor environments, such as minimizing biomass use and better ventilation. Whereas we did not assess outdoor air quality, studies elsewhere indicate that poor outdoor air quality could aggravate/increase the risk of respiratory symptoms [39, 40]. Further studies should also consider the role of outdoor pollution on respiratory morbidity.

We also found reduced risk of shortness of breath and coughing among adults in households whose cooking place was outside their living house than those who cooked from inside their living spaces. This could be attributed to the fact that outside cooking is protective as indoors cooking with the poor ventilation in the slums facilitates the pollutant accumulation from the cooking fuels, consequently impairing respiratory system. When cooking occurs outside the house or in an open area, the cooking smoke dissipates quickly, reducing exposure to individuals and lowering the risk of respiratory problems. Previous studies have documented a higher concentration of these air pollutants in households where indoor cooking takes place [41] However, it is important to note that our study did not find statistically significant differences in pollutant levels. Our findings reaffirm those reported in studies in Thailand and Ethiopia which indicated that cooking inside a home was predictive of higher risk of respiratory symptoms, such as dyspnea (shortness of breath) [42, 43]. Therefore, there is a need to increase the sensibilization of residents of slum settlements on the health risks associated with cooking indoors, especially when using unclean fuels such as firewood and charcoal.

Our findings indicate that owning pets in the household was associated with increased likelihood of cough among adults and wheezing among children. Allergens from domestic pets, such as cats, dogs, and birds, can trigger sensitivities in individuals who are predisposed to allergies and cause respiratory problems [44]. Therefore, individuals who are susceptible to these allergens are advised to either avoid rearing these pets in their homes or take necessary precautions to minimize exposure to allergens. Additionally, we observed an association between indoor insecticide spraying (IRS) and shortness of breath in adults and phlegm in children. Although IRS is a control measure for disease vectors, including mosquitoes and other vectors, it emits various indoor air pollutants, including hazardous air pollutants and VOCs, which can settle on surfaces, furniture and counters and can also be inhaled by occupants of the houses. Overuse or incorrect use leads to the build-up of residues that may contribute to respiratory health problems when inhaled. A direct relationship between respiratory symptoms in children and exposure to insecticides has been demonstrated elsewhere [45]. Our findings indicate a need to emphasize and promote non-chemical insect/pest control methods in informal settlements to reduce exposure to harmful chemical pollutants. Ensuring enough time is allowed between spraying and occupation of the sprayed room can also minimise the residual effect, lowering the risk of respiratory problems.

Dampness was found to be associated with symptoms such as phlegm and shortness of breath in adults, as well as cough and phlegm in children. Nearly half of the households were characterised by indoor dampness which is not surprising since Bwaise frequently floods and has poor drainage. Dampness creates an environment for mould spores to grow, which can trigger allergic reactions and respiratory problems. In addition, existing evidence indicates that dampness can encourage chemical or biological degradation of materials and thus increase the indoor concentration of pollutants which could increase the risk of respiratory problems [25]. Extant research in developed countries also indicates a high likelihood of phlegm among household occupants in damp and moist dwellings [46, 47]. Although there is limited evidence of the association between residential dampness and phlegm in adults, Simoni and colleagues, in a study conducted in Italy, previously reported that dampness increased the risk of phlegm in children [48].

### Strengths and limitations

This is among Uganda’s first studies to explore the associations between indoor air conditions and respiratory health among adults and children. Nevertheless, our study had some limitations regarding the design and interpretation of findings. This was a cross-sectional study in which causality between exposure to indoor air pollution and respiratory outcomes cannot be confirmed since we cannot determine temporality. Our measurement of air quality parameters was limited to 5 hours of same-day measurement and not 24 hours. However, we captured the most active period when most households would at least do some cooking, and at least 10 measurements (3-minute averages each) were made at each household throughout the monitoring. There is a need to use prospective analytical study designs with longer duration of measurements to examine the hypotheses further and determine the predictors of respiratory problems among urban slum dwellers. In addition, the study was subject to recall bias and subjectivity as we relied on self-reports in assessing respiratory problems and other indoor air conditions. Few households were using cleaner fuels, making it difficult to study the impact on respiratory outcomes.

## Conclusions

Our study supports evidence that poor indoor air conditions can adversely affect the respiratory health of adults and children in informal urban settings. Our findings emphasize the importance of identifying and developing interventions to reduce environmental triggers through simple home modifications and household education. We suggest that residents must limit time indoors and ensure sufficient ventilation. We also recommend additional prospective studies with longer-duration of pollutant measurements are necessary to characterize the dose-response relationships between the various home air pollutants and respiratory symptoms.

## Supporting information

S1 Fig Temperature and humidity changes. (DOCX)

S1 Table Distribution of respiratory outcomes in adults. (DOCX)

S2 Table Distribution of respiratory outcomes in children. (DOCX)

S1 Text DAGs and univariable analysis. (DOCX)

S2 Text PLOS questionnaire on inclusivity in global health. (DOCX)

S1 Data Dataset for adults information. (CSV)

S2 Data Dataset for children information. (CSV)
