# Supplementary material for: Indoor air pollutants and respiratory symptoms among residents of an informal urban settlement in Uganda: A cross-sectional study
Source: PLoS One. 2023 Aug 17;18(8):e0290170. doi: 10.1371/journal.pone.0290170 (PMC10434877; doi:10.1371/journal.pone.0290170)
Supplement: S1 Fig — (DOCX) [file pone.0290170.s001.docx]

S6 Figure. Temperature and Humidity changes during data collection period.

Average Temperature over data collection days

Average relative humidity
