# Supplementary material for: Indoor air pollutants and respiratory symptoms among residents of an informal urban settlement in Uganda: A cross-sectional study
Source: PLoS One. 2023 Aug 17;18(8):e0290170. doi: 10.1371/journal.pone.0290170 (PMC10434877; doi:10.1371/journal.pone.0290170)
Supplement: S1 Table — (DOCX) [file pone.0290170.s002.docx]

S2 Table: Distribution of respiratory outcomes in adults by participant characteristics

|  |  | **Cough** | | **Phlegm** | | **Wheezing** | | **Running nose** | | **Shortness of breath** | |
| --- | --- | --- | --- | --- | --- | --- | --- | --- | --- | --- | --- |
| **Characteristics** | **TOTAL** | **No** | **Yes** | **No** | **Yes** | **No** | **Yes** | **No** | **Yes** | **No** | **Yes** |
| **Gender** |  |  |  |  |  |  |  |  |  |  |  |
| Female | 242(85.2%) | 85(88.5%) | 157(83.5%) | 201(85.9%) | 41(82.0%) | 202(83.5%) | 40(95.2%) | 160(84.7%) | 82(86.3%) | 138(83.6%) | 104(87.4%) |
| Male | 42(14.8%) | 11(11.5%) | 31(16.5%) | 33(14.1%) | 9(18.0%) | 40(16.5%) | 2(4.8%) | 29(15.3%) | 13(13.7%) | 27(16.4%) | 15(12.6%) |
| **Age in years** |  |  |  |  |  |  |  |  |  |  |  |
| < 30 years | 146(51.4%) | 50(52.1%) | 96(51.1%) | 121(51.7%) | 25(50.0%) | 130(53.7%) | 16(38.1%) | 97(51.3%) | 49(51.6%) | 79(47.9%) | 67(56.3%) |
| 30 - 45 | 108(38.0%) | 32(33.3%) | 76(40.4%) | 88(37.6%) | 20(40.0%) | 85(35.1%) | 23(54.8%) | 70(37.0%) | 38(40.0%) | 62(37.6%) | 46(38.7%) |
| > 45 | 30(10.6%) | 14(14.6%) | 16(8.5%) | 25(10.7%) | 5(10.0%) | 27(11.2%) | 3(7.1%) | 22(11.6%) | 8(8.4%) | 24(14.5%) | 6(5.0%) |
| **Marital status** |  |  |  |  |  |  |  |  |  |  |  |
| Married | 136(47.9%) | 52(54.2%) | 84(44.7%) | 118(50.4%) | 18(36.0%) | 125(51.7%) | 11(26.2%) | 96(50.8%) | 40(42.1%) | 82(49.7%) | 54(45.4%) |
| Separated | 43(15.1%) | 16(16.7%) | 27(14.4%) | 36(15.4%) | 7(14.0%) | 36(14.9%) | 7(16.7%) | 30(15.9%) | 13(13.7%) | 20(12.1%) | 23(19.3%) |
| Single | 105(37.0%) | 28(29.2%) | 77(41.0%) | 80(34.2%) | 25(50.0%) | 81(33.5%) | 24(57.1%) | 63(33.3%) | 42(44.2%) | 63(38.2%) | 42(35.3%) |
| **Education** |  |  |  |  |  |  |  |  |  |  |  |
| None | 25(8.8%) | 7(7.3%) | 18(9.6%) | 21(9.0%) | 4(8.0%) | 18(7.4%) | 7(16.7%) | 16(8.5%) | 9(9.5%) | 16(9.7%) | 9(7.6%) |
| Primary | 114(40.1%) | 36(37.5%) | 78(41.5%) | 89(38.0%) | 25(50.0%) | 98(40.5%) | 16(38.1%) | 73(38.6%) | 41(43.2%) | 71(43.0%) | 43(36.1%) |
| Post primary | 145(51.1%) | 53(55.2%) | 92(48.9%) | 124(53.0%) | 21(42.0%) | 126(52.1%) | 19(45.2%) | 100(52.9%) | 45(47.4%) | 78(47.3%) | 67(56.3%) |
| **Occupation** |  |  |  |  |  |  |  |  |  |  |  |
| Employed | 195(68.7%) | 59(61.5%) | 136(72.3%) | 157(67.1%) | 38(76.0%) | 163(67.4%) | 32(76.2%) | 123(65.1%) | 72(75.8%) | 119(72.1%) | 76(63.9%) |
| Other | 17(6.0%) | 7(7.3%) | 10(5.3%) | 14(6.0%) | 3(6.0%) | 13(5.4%) | 4(9.5%) | 15(7.9%) | 2(2.1%) | 5(3.0%) | 12(10.1%) |
| Unemployed | 72(25.4%) | 30(31.3%) | 42(22.3%) | 63(26.9%) | 9(18.0%) | 66(27.3%) | 6(14.3%) | 51(27.0%) | 21(22.1%) | 41(24.8%) | 31(26.1%) |
| **Cooking place location** | |  |  |  |  |  |  |  |  |  |  |
| Inside | 90(31.7%) | 26(27.1%) | 64(34.0%) | 69(29.5%) | 21(42.0%) | 74(30.6%) | 16(38.1%) | 49(25.9%) | 41(43.2%) | 54(32.7%) | 36(30.3%) |
| Outside | 194(68.3%) | 70(72.9%) | 124(66.0%) | 165(70.5%) | 29(58.0%) | 168(69.4%) | 26(61.9%) | 140(74.1%) | 54(56.8%) | 111(67.3%) | 83(69.7%) |
| **Income in USD** | |  |  |  |  |  |  |  |  |  |  |
| < 50 | 77(27.1%) | 29(30.2%) | 48(25.5%) | 67(28.6%) | 10(20.0%) | 68(28.1%) | 9(21.4%) | 53(28.0%) | 24(25.3%) | 46(27.9%) | 31(26.1%) |
| 50 - 150 | 163(57.4%) | 51(53.1%) | 112(59.6%) | 127(54.3%) | 36(72.0%) | 136(56.2%) | 27(64.3%) | 104(55.0%) | 59(62.1%) | 101(61.2%) | 62(52.1%) |
| > 150 | 44(15.5%) | 16(16.7%) | 28(14.9%) | 40(17.1%) | 4(8.0%) | 38(15.7%) | 6(14.3%) | 32(16.9%) | 12(12.6%) | 18(10.9%) | 26(21.8%) |
| **PM 2.5^1^** | 0.39(0.34, 0.45) | 0.37(0.34, 0.42) | 0.40(0.35,0.45) | 0.39(0.34, 0.44) | 0.40(0.36, 0.46) | 0.39(0.34, 0.45) | 0.39(0.35, 0.45) | 0.39(0.34, 0.44) | 0.39(0.35, 0.45) | 0.39(0.34, 0.45) | 0.39(0.35, 0.43) |
| **Carbon monoxide** | 8 (4, 12) | 7 (5, 12) | 8 (4, 13) | 8 (5, 12) | 8 (1, 16) | 8 (5, 14) | 7 (2, 10) | 8 (5, 14) | 7 (2, 11) | 7 (4, 13) | 8 (4, 12) |
| **Main fuel type** |  |  |  |  |  |  |  |  |  |  |  |
| Non-biomass | 15(5.3%) | 9(9.4%) | 6(3.2%) | 14(6.0%) | 1(2.0%) | 14(5.8%) | 1(2.4%) | 10(5.3%) | 5(5.3%) | 8(4.8%) | 7(5.9%) |
| Biomass | 269(94.7%) | 87(90.6%) | 182(96.8%) | 220(94.0%) | 49(98.0%) | 228(94.2%) | 41(97.6%) | 179(94.7%) | 90(94.7%) | 157(95.2%) | 112(94.1%) |
| **Pets** |  |  |  |  |  |  |  |  |  |  |  |
| No | 259(91.2%) | 93(96.9%) | 166(88.3%) | 215(91.9%) | 44(88.0%) | 223(92.1%) | 36(85.7%) | 174(92.1%) | 85(89.5%) | 149(90.3%) | 110(92.4%) |
| Yes | 25(8.8%) | 3(3.1%) | 22(11.7%) | 19(8.1%) | 6(12.0%) | 19(7.9%) | 6(14.3%) | 15(7.9%) | 10(10.5%) | 16(9.7%) | 9(7.6%) |
| **Carpets in house** | |  |  |  |  |  |  |  |  |  |  |
| No | 157(55.3%) | 49(51.0%) | 108(57.4%) | 126(53.8%) | 31(62.0%) | 129(53.3%) | 28(66.7%) | 100(52.9%) | 57(60.0%) | 92(55.8%) | 65(54.6%) |
| yes | 127(44.7%) | 47(49.0%) | 80(42.6%) | 108(46.2%) | 19(38.0%) | 113(46.7%) | 14(33.3%) | 89(47.1%) | 38(40.0%) | 73(44.2%) | 54(45.4%) |
| Dampness |  |  |  |  |  |  |  |  |  |  |  |
| No | 161(56.7%) | 58(60.4%) | 103(54.8%) | 145(62.0%) | 16(32.0%) | 142(58.7%) | 19(45.2%) | 120(63.5%) | 41(43.2%) | 93(56.4%) | 68(57.1%) |
| Yes | 123(43.3%) | 38(39.6%) | 85(45.2%) | 89(38.0%) | 34(68.0%) | 100(41.3%) | 23(54.8%) | 69(36.5%) | 54(56.8%) | 72(43.6%) | 51(42.9%) |
| **Indoor residual spraying** | |  |  |  |  |  |  |  |  |  |  |
| No | 196(69.0%) | 72(75.0%) | 124(66.0%) | 163(69.7%) | 33(66.0%) | 166(68.6%) | 30(71.4%) | 138(73.0%) | 58(61.1%) | 110(66.7%) | 86(72.3%) |
| Yes | 88(31.0%) | 24(25.0%) | 64(34.0%) | 71(30.3%) | 17(34.0%) | 76(31.4%) | 12(28.6%) | 51(27.0%) | 37(38.9%) | 55(33.3%) | 33(27.7%) |
| **Smoking** |  |  |  |  |  |  |  |  |  |  |  |
| No | 243(85.6%) | 87(90.6%) | 156(83.0%) | 203(86.8%) | 40(80.0%) | 206(85.1%) | 37(88.1%) | 161(85.2%) | 82(86.3%) | 140(84.8%) | 103(86.6%) |
| Yes | 41(14.4%) | 9(9.4%) | 32(17.0%) | 31(13.2%) | 10(20.0%) | 36(14.9%) | 5(11.9%) | 28(14.8%) | 13(13.7%) | 25(15.2%) | 16(13.4%) |

Note: ^1^1/10 of log transformed PM2.5
