# Supplementary material for: Indoor air pollutants and respiratory symptoms among residents of an informal urban settlement in Uganda: A cross-sectional study
Source: PLoS One. 2023 Aug 17;18(8):e0290170. doi: 10.1371/journal.pone.0290170 (PMC10434877; doi:10.1371/journal.pone.0290170)
Supplement: S2 Table — (DOCX) [file pone.0290170.s003.docx]

S3 Table: Distribution of respiratory outcomes in children by participant characteristics

|  |  | **Cough** | | **Phlegm** | | **Wheezing** | | **Running nose** | | **Shortness of breath** | |
| --- | --- | --- | --- | --- | --- | --- | --- | --- | --- | --- | --- |
| **Characteristics** | **TOTAL** | **No** | **Yes** | **No** | **Yes** | **No** | **Yes** | **No** | **Yes** | **No** | **Yes** |
| Childs sex |  |  |  |  |  |  |  |  |  |  |  |
| Female | 121(52.6%) | 72(47.7%) | 49(62.0%) | 105(52.8%) | 16(51.6%) | 96(52.2%) | 25(54.3%) | 63(49.6%) | 58(56.3%) | 88(52.1%) | 33(54.1%) |
| Male | 109(47.4%) | 79(52.3%) | 30(38.0%) | 94(47.2%) | 15(48.4%) | 88(47.8%) | 21(45.7%) | 64(50.4%) | 45(43.7%) | 81(47.9%) | 28(45.9%) |
| Age in years |  |  |  |  |  |  |  |  |  |  |  |
| < 2 | 106(46.9%) | 74(50.3%) | 32(40.5%) | 91(46.7%) | 15(48.4%) | 86(47.8%) | 20(43.5%) | 61(49.6%) | 45(43.7%) | 77(46.7%) | 29(47.5%) |
| 2+ | 120(53.1%) | 73(49.7%) | 47(59.5%) | 104(53.3%) | 16(51.6%) | 94(52.2%) | 26(56.5%) | 62(50.4%) | 58(56.3%) | 88(53.3%) | 32(52.5%) |
| Education |  |  |  |  |  |  |  |  |  |  |  |
| Not in school | 166(72.2%) | 112(74.2%) | 54(68.4%) | 145(72.9%) | 21(67.7%) | 131(71.2%) | 35(76.1%) | 90(70.9%) | 76(73.8%) | 119(70.4%) | 47(77.0%) |
| Schooling | 64(27.8%) | 39(25.8%) | 25(31.6%) | 54(27.1%) | 10(32.3%) | 53(28.8%) | 11(23.9%) | 37(29.1%) | 27(26.2%) | 50(29.6%) | 14(23.0%) |
| Cooking place location | |  |  |  |  |  |  |  |  |  |  |
| Inside | 73(31.7%) | 49(32.5%) | 24(30.4%) | 59(29.6%) | 14(45.2%) | 54(29.3%) | 19(41.3%) | 34(26.8%) | 39(37.9%) | 49(29.0%) | 24(39.3%) |
| Outside | 157(68.3%) | 102(67.5%) | 55(69.6%) | 140(70.4%) | 17(54.8%) | 130(70.7%) | 27(58.7%) | 93(73.2%) | 64(62.1%) | 120(71.0%) | 37(60.7%) |
| PM 2.5^1^ |  | 0.40 (0.34, 0.44) | 0.39 (0.35, 0.43) | 0.39 (0.34, 0.43) | 0.41(0.38, 0.48) | 0.39(0.34, 0.44) | 0.39(0.34, 0.45) | 0.40(0.35, 0.45) | 0.38(0.34, 0.42) | 0.39(0.34, 0.44) | 0.40(0.34, 0.44) |
| Carbon monoxide | 8 (5, 12) | 8 (5, 12) | 8 (4, 12) | 8 (5, 12) | 9 (6, 14) | 8 (5, 12) | 8 (4, 11) | 8 (4, 13) | 8 (6, 12) | 8 (5, 12) | 9 (5, 16) |
| Main fuel type |  |  |  |  |  |  |  |  |  |  |  |
| Non-biomass | 9(3.9%) | 7(4.6%) | 2(2.5%) | 9(4.5%) | 0(0.0%) | 8(4.3%) | 1(2.2%) | 6(4.7%) | 3(2.9%) | 7(4.1%) | 2(3.3%) |
| Biomass | 221(96.1%) | 144(95.4%) | 77(97.5%) | 190(95.5%) | 31(100.0%) | 176(95.7%) | 45(97.8%) | 121(95.3%) | 100(97.1%) | 162(95.9%) | 59(96.7%) |
| Pets |  |  |  |  |  |  |  |  |  |  |  |
| No | 201(87.4%) | 134(88.7%) | 67(84.8%) | 175(87.9%) | 26(83.9%) | 164(89.1%) | 37(80.4%) | 106(83.5%) | 95(92.2%) | 147(87.0%) | 54(88.5%) |
| Yes | 29(12.6%) | 17(11.3%) | 12(15.2%) | 24(12.1%) | 5(16.1%) | 20(10.9%) | 9(19.6%) | 21(16.5%) | 8(7.8%) | 22(13.0%) | 7(11.5%) |
| Carpets in house | |  |  |  |  |  |  |  |  |  |  |
| No | 136(59.1%) | 91(60.3%) | 45(57.0%) | 116(58.3%) | 20(64.5%) | 109(59.2%) | 27(58.7%) | 73(57.5%) | 63(61.2%) | 102(60.4%) | 34(55.7%) |
| yes | 94(40.9%) | 60(39.7%) | 34(43.0%) | 83(41.7%) | 11(35.5%) | 75(40.8%) | 19(41.3%) | 54(42.5%) | 40(38.8%) | 67(39.6%) | 27(44.3%) |
| Dampness |  |  |  |  |  |  |  |  |  |  |  |
| No | 125(54.3%) | 89(58.9%) | 36(45.6%) | 123(61.8%) | 2(6.5%) | 106(57.6%) | 19(41.3%) | 74(58.3%) | 51(49.5%) | 96(56.8%) | 29(47.5%) |
| Yes | 105(45.7%) | 62(41.1%) | 43(54.4%) | 76(38.2%) | 29(93.5%) | 78(42.4%) | 27(58.7%) | 53(41.7%) | 52(50.5%) | 73(43.2%) | 32(52.5%) |
| Indoor residual spraying | |  |  |  |  |  |  |  |  |  |  |
| No | 151(65.7%) | 94(62.3%) | 57(72.2%) | 139(69.8%) | 12(38.7%) | 123(66.8%) | 28(60.9%) | 69(54.3%) | 82(79.6%) | 109(64.5%) | 42(68.9%) |
| Yes | 79(34.3%) | 57(37.7%) | 22(27.8%) | 60(30.2%) | 19(61.3%) | 61(33.2%) | 18(39.1%) | 58(45.7%) | 21(20.4%) | 60(35.5%) | 19(31.1%) |
| Smoking |  |  |  |  |  |  |  |  |  |  |  |
| No | 193(83.9%) | 123(81.5%) | 70(88.6%) | 166(83.4%) | 27(87.1%) | 155(84.2%) | 38(82.6%) | 109(85.8%) | 84(81.6%) | 141(83.4%) | 52(85.2%) |
| Yes | 37(16.1%) | 28(18.5%) | 9(11.4%) | 33(16.6%) | 4(12.9%) | 29(15.8%) | 8(17.4%) | 18(14.2%) | 19(18.4%) | 28(16.6%) | 9(14.8%) |

Note: ^1^1/10 of log transformed PM2.5
