# Supplementary material for: Indoor air pollutants and respiratory symptoms among residents of an informal urban settlement in Uganda: A cross-sectional study
Source: PLoS One. 2023 Aug 17;18(8):e0290170. doi: 10.1371/journal.pone.0290170 (PMC10434877; doi:10.1371/journal.pone.0290170)
Supplement: S1 Text — (DOCX) [file pone.0290170.s004.docx]

**S1. Text. DAGs and univariable analysis**

1. **DAG FOR SELECTION OF VARIABLES**

**DAG for adults**

Marital status

Occupation

Education

Length of stay in settlement

Income

Pet raring

PM2.5, PM10, C0

Dampness,

IRS

Respiratory Problems

Age

Gender

Fuel type

Cooking place

Smoking

**DAG for children**

Marital status

Parental Occupation

Education

Length of stay

Income

Pet raring

PM2.5, PM10, C0

Dampness,

IRS

Respiratory Problems

Age

Gender

Fuel type

Cooking place

Smoking

1. **Bivariable analyses**

**Table I: Bivariate analysis of the factors associated with respiratory problems among adults**

| **Attributes** | **Cough**  PR (95% CI) | **Phlegm**  PR (95% CI) | **Wheezing**  PR (95% CI) | **Blocked /Runny nose**  PR (95% CI) | **Shortness of breath**  PR (95% CI) |
| --- | --- | --- | --- | --- | --- |
| **Demographic characteristics** |  |  |  |  |  |
| **Gender: Male** | 1.14 (0.77 – 1.67) | 1.26 (0.61 – 2.60) | 0.29 (0.07 – 1.19) | 0.83 (0.48 – 1.42) | 0.91 (0.51 – 1.63) |
| Age (in years) |  |  |  |  |  |
| < 30 | 1 | 1 | 1 | 1 | 1 |
| 30 – 45 | 1.07 (0.90 – 1.27) | 1.08 (0.63 – 1.84) | 1.94 (1.07 – 3.50) | 0.93 (0.70 – 1.23) | 1.04 (0.74 – 1.48) |
| 45 + | 0.81 (0.56 – 1.16) | 0.97 (0.40 – 2.34) | 0.91 (0.28 – 2.94) | 0.43 (0.21 – 0.91) | 0.79 (0.42 – 1.50) |
| **Marital status** |  |  |  |  |  |
| Married | 1 | 1 | 1 | 1 | 1 |
| Separated | 1.02 (0.66 – 1.57) | 1.22 (0.51 – 294) | 2.01 (0.78 – 5.19) | 1.34 (0.82 – 2.19) | 1.03 (0.55 – 1.92) |
| Single | 1.19 (0.87 – 1.62) | 1.79 (0.98 – 3.30) | 2.83 (1.38 – 5.76) | 1.01 (0.67 – 1.51) | 1.36 (0.88 – 2.07) |
| **Education level** |  |  |  |  |  |
| Non-formal | 1 | 1 | 1 | 1 | 1 |
| Primary | 0.95 (0.57 – 1.59) | 1.37 (0.48 – 3.94) | 0.5ß (0.21 – 1.21) | 1.05 (0.51 – 2.14) | 1.00 (0.49 – 2.06) |
| Post-primary | 0.88 (0.53 – 1.46) | 0.91 (0.31 – 2.64) | 0.47 (0.20 – 1.11) | 1.28 (0.64 – 2.57) | 0.86 (0.42 – 1.76) |
| **Occupation** |  |  |  |  |  |
| Employed (including business) | 1 | 1 | 1 | 1 | 1 |
| Unemployed | 0.84 (0.56 – 1.26) | 0.90 (0.31 – 2.63) | 1.43 (0.57 – 3.58) | 1.81 (1.27 – 2.58) | 0.31 (0.10 – 1.19) |
| Other | 0.84 (0.67 – 1.04) | 064 (0.33 – 1.26) | 0.51 (0.22 – 1.17) | 1.10 (0.80 – 1.51) | 0.78 (0.52 – 1.18) |
| **Housing characteristics** |  |  |  |  |  |
| **Owner of dwelling: No** | 1.36 (0.77 – 2.38) | 1.15 (0.42 – 3.21) | 1.32 (0.40 – 4.23) | 1.62 (0.75 – 3.46) | 1.27 (0.59 – 2.74) |
| **HH Income** |  |  |  |  |  |
| < 50 | 1 | 1 | 1 | 1 | 1 |
| 50 – 150 | 1.10 (0.79 – 1.54) | 1.70 (0.84 – 3.42) | 1.41 (0.67 – 3.01) | 0.94 (0.61 – 1.45) | 1.16 (0.72 – 1.87) |
| >150 | 1.02 (0.64 – 1.62) | 0.70 (0.22 – 2.23) | 1.17 (0.42 – 3.27) | 1.47 (0.87 – 2.47) | 0.88 (0.43 – 1.75) |
| **Cooking outside the living house** | 0.90 (0.66 – 1.21) | 0.64 (0.37 – 1.12) | 0.75 (0.40 – 1.41) | 1.06 (0.72 – 1.58) | 0.61 (0.41 – 0.92) |
| Humidity | 1.01 (0.98 – 1.04) | 1.04 (0.99 – 1.10) | 1.05 (0.99 – 1.12) | 1.02 (0.98 – 1.06) | 1.02 (0.97 – 1.06) |
| Temperature | 0.98 (0.87 – 1.12) | 0.82 (0.65 – 1.06) | 0.91 (0.69 – 1.21) | 0.92 (0.78 – 1.08) | 0.92 (0.77 – 1.11) |
| PM 2.5^1^ | 3.90 (1.37 – 11.18) | 9.21 (0.33 – 269.27)) | 2.29 (0.03 – 161.56) | 0.48 (0.07 – 3.38) | 2.02 (0.20 – 20.48) |
| PM10^2^ | 3.76 (1.24 – 11.40)) | 7.81 (0.23– 261.52)) | 1.62 (0.02 – 142.06)) | 0.44 (0.06 – 3.26) | 2.37 (0.22 – 25.38) |
| Carbon monoxide | 1.00 (0.95 – 1.07) | 1.07 (0.91 – 1.26)) | 0.65 (0.46– 0.91)) | 1.01 (0.93 – 1.12) | 0.90 (0.76 – 1.06) |
| Main fuel: cleaner fuel vs biomass | 0.59 (0.26 – 1.33) | 0.37 (0.05 – 2.65) | 0.44 (0.06 – 3.17) | 1.12 (0.52 – 2.41) | 1.00 (0.41 – 2.45) |
| Rearing pets | 1.37 (0.88 – 2.14) | 1.41 (0.60 – 3.31) | 1.72 (0.73 – 4.09) | 0.84 (0.42 – 1.67) | 1.22 (0.63 – 2.34) |
| Carpets in the living room | 0.92 (0.69 – 1.22) | 0.75 (0.43 – 1.34) | 0.61 (0.32 – 1.17) | 1.02 (0.72 – 1.47) | 0.82 (0.55 – 1.24) |
| Home dampness | 1.08 (0.81 – 1.44) | 2.78 (1.53 – 5.01) ^*^ | 1.58 (0.86 – 2.91) | 0.98 (0.68 – 1.41) | 1.72 (1.15 – 2.58) |
| Smoker | 1.22 (0.83 – 1.78) | 1.48 (0.74 – 2.96) | 0.80 (0.31 – 2.03) | 0.92 (0.54 – 1.56) | 0.94 (0.52 – 1.69) |
| Indoor spraying for insecticides | 1.15 (0.85 – 1.55) | 1.14 (0.64 – 2.06) | 0.89 (0.46 – 1.74) | 0.85 (0.57 – 1.28) | 1.42 (0.94 – 2.14) |

Note: ^1^ PM2.5; 1/10 of log transformed PM2.5 average values; ^2^ PM10; 1/10 of log transformed PM10 average values

Table II: Bivariate analysis of the factors associated with self-reported respiratory problems among children.

| **Attributes** | **Morning Cough** | **Day or night cough** | **Phlegm** | **Wheezing** | **Blocked / Runny nose** | **Shortness of breath** |
| --- | --- | --- | --- | --- | --- | --- |
| **Demographic characteristics** | |  |  |  |  |  |
| **Gender:** Male | 1.09 (0.81-1.45) | 0.68 (0.43 – 1.07) | 1.04 (0.51 – 2.10) | 0.93 (0.52 – 1.67) | 0.86 (0.58 – 1.27) | 0.94 (0.57 – 1.56) |
| **Age (in years)** |  |  |  |  |  |  |
| **<= 2** | 1 | 1 | 1 | 1 | 1 | 1 |
| **2+** | 1.00 (0.87 – 1.13) | 0.98 (0.68 – 1.40) | 0.91 (0.47 – 1.76) | 1.11 (0.66 – 1.86) | 1.04 (0.79 – 1.40) | 0.94 (0.61 – 1.45) |
| **Child Education** |  |  |  |  |  |  |
| Not in school | 1 | 1 | 1 | 1 | 1 | 1 |
| School | 1.05 (0.76 – 1.44) | 1.20 (0.75 – 1.93) | 1.24 (0.58 – 2.62) | 0.82 (0.41 – 1.61) | 0.92 (0.59 – 1.43) | 0.77 (0.42 – 1.40) |
| **Housing conditions** |  |  |  |  |  |  |
| **Cooking place: Outside** | 1.04 (0.76 – 1.42) | 1.07 (0.66 – 1.72) | 0.56 (0.28 – 1.14) | 0.66 (0.37 – 1.19) | 0.76 (0.51 – 1.14) | 0.72 (0.43 – 1.20) |
| PM2.5 | 1.14 (0.43 – 3.04) | 0.39 (0.03 – 4.50) | 289.6 (4.86 – 17229.61) | 2.56 (0.05 – 121.91) | 0.47 (0.05 – 4.06) | 0.90(0.05– 16.91) |
| Carbon monoxide | 1.05 (0.92 - 1.20) | 0.91 (0.63 – 130) | 1.23 (0.63 – 2.39) | 1.05 (0.63 – 1.77) | 0.98 (0.73 1.30) | 1.19 (0.77– 1.84) |
| **Main fuel:** Biomass | 0.90 (0.44 – 1.82) | 1.56 (0.39 – 6.38) |  | 1.83 (0.25 – 13.29) | 1.36 (0.43 – 4.28) | 1.20 (0.29 – 4.92) |
| **Rearing pets** | 0.89 (0.57 – 1.41) | 1.24 (0.67 – 2.29) | 1.33 (0.51 – 3.47) | 1.69 (0.81 – 3.49) | 0.58 (0.28 – 1.21) | 0.90 (0.41 – 1.97) |
| **Carpets in the living room** | 1.06 (0.79 – 1.43) | 1.09 (0.70 – 1.71) | 0.80 (0.38 – 1.66) | 1.02 (0.57 – 1.83) | 0.92 (0.62 – 1.36) | 1.14 (0.69 – 1.90) |
| **Home dampness** | 1.07 (0.80 – 1.43) | 1.42 (0.91 – 2.21) | 17.26 (4.11 – 72.34) | 1.69 (0.94 – 3.04) | 1.21 (0.82 – 1.79) | 1.31 (0.79 – 2.17) |
| **Parent / guardian smokes** | 0.98 (0.66 – 1.45) | 0.67 (0.34 – 1.34) | 0.77 (0.27 – 2.20) | 1.10 (0.51 – 2.35) | 1.18 (0.72 – 1.94) | 0.90 (0.44 – 1.83) |
| **Indoor spraying for insecticides** | 1.04 (0.77 – 1.41) | 0.74 (0.45 – 1.21) | 3.03 (1.47 – 6.23) | 1.23 (0.68 – 2.22) | 2.04 (1.26 – 3.29) | 0.86 (0.50 – 1.48) |

Note: ^1^ PM2.5; 1/10 of log transformed PM2.5 average values
