# Supplementary material for: Indoor air pollutants and respiratory symptoms among residents of an informal urban settlement in Uganda: A cross-sectional study
Source: PLoS One. 2023 Aug 17;18(8):e0290170. doi: 10.1371/journal.pone.0290170 (PMC10434877; doi:10.1371/journal.pone.0290170)
Supplement: Attachment — Submitted filename: Response to IAQ comments_27052023.docx [file pone.0290170.s008.docx]

Makerere University

School of Public Health

P.O Box 7072, Kampala

27^th^ May 2023

The Editor,

PLOS ONE Journal.

**Re: Submission of a revised review article for publication**

We would like to express our gratitude to you for providing us with the opportunity to revise our manuscript titled “***Indoor air pollutants and respiratory symptoms among residents of an informal urban settlement in Uganda: a cross-sectional study”.***  We are also thankful to the peer reviewers for their valuable insights, which have significantly enhanced the quality of our work.

In response to the reviewers' comments, we have made diligent efforts to address each point raised, striving to incorporate their suggestions and improve the manuscript accordingly. We are now pleased to submit the revised version of our manuscript for your consideration.

Sincerely,

Solomon Wafula

**#General comments**

Statistical analysis needs to be revised. In particular:

1) an analysis using exposure on the continuous scale should be performed, or a valid reason not to do it should be provided (Reviewer 2)

We have now performed re-analysis using exposure on the continuous scale where necessary in the revised manuscript. A detailed response is under specific comments.

2) the multivariable analysis should be conducted on an a-priori set of confounders, not basing on data-driven strategies (Reviewer 2).

Thank you for the suggestion. We have conducted the multivariable analysis on an a-priori set of confounders based on the DAG theory and we provide rationale for the choice of priori set of confounders in the methods.

3) the use of p-value categorization should be avoided (Reviewer 2)

**Response**: We have avoided the use of p-value categorization in the revised manuscript as per your suggestion.

Some additional information needs to be provided. In particular:

1) Number of inhabitants in the different Bwaise districts (Reviewer 1/ Reviewer 2)

We have provided the number of inhabitants in the different Bwaise zones…. on page 4

2) proportion of non-respondents (Reviewer 1)

Thank you for the suggestion but in this study, we did not have any non-response.

Please also consider these additional comments from my side:

In table 2, the line about main fuel type is not clear. I assume that about 95% of household used biomass. Please correct the table accordingly.

We have corrected table 2 to make the line about main fuel type clearer.

According to the methods section, all children under five years in the selected households were included in the analysis. However, in the results section is reported that analysis was carried out on 230 children out of 284 households, less than one per household. Could you please double-check this number?

Thank you for the comment. We understand that the number of children included in the analysis is lower than expected but we confirm that this number is correct. We found fewer children (*n* = 230) than households (*n* = 284) as many households had no children. Having a child under 5 was not a requirement for a household to be included in the study.

In the results section, you reported that prevalence of wheezing was 2.26 higher among single participants compared to married ones. However, this is the result of the univariable analysis, which is not adjusted for age, gender, smoking, etc. if you want to analyze this association (even if it is out of the main scope of the paper), you should report the adjusted results.

Thank you for your observation. We have now reported the updated adjusted results. However, since the study focused on indoor air conditions and respiratory problem, we do not provide narratives for the association between socio-demographic variables and respiratory problems.

Apparently, the paragraph titled “Associations between indoor air conditions and respiratory problems among parent-reported respiratory problems among children” include only one sentence. It should be merged with the following one.

We have merged the two paragraphs combining the narratives from bivariate analysis and multivariable analysis

**Minor issues**

**Comment**: At line 234 you may want to say “the effect of fuel type” instead of “The predictive value of fuel type”

**Response**: We have corrected the wording to read “the effect of fuel type” instead of “The predictive value of fuel type.”

**Comment**: Line 246 “…and the finding is consistent” please cancel “and”

**Response**: We have removed “and” from line 246 for clarity.

**Comment**: Line 247 for sake of clarity, you may want to say “Indoor PM concentration is related to inflammation and a decrease in lung function”

**Response**: We have revised the wording at line 247 to read “Indoor PM concentration is related to inflammation and decreased lung function” for clarity.

**Comment**: Line 264 please use sensibilization instead of sensitization

**Response:** We have used “sensibilization” instead of “sensitization” in line 264.

**Comment**: Line 287 please refer to “Simoni and colleagues”

**Response**: We have referred to “Simoni et al.” at line 287

Reviewer #1: This paper is one of the first aimed at providing some information on the effects of environmental pollution on the health of residents in Uganda. Some findings (eg the protective effect of outdoor cooking) are convincing and may have an impact on public health policies.
The authors honestly recognize the limitations of their study.

**Response:** Thank you for reviewing our manuscript and providing constructive feedback. We appreciate the time and effort you have taken to read and evaluate our study. Below is our point-by-point response to your comments:

Thus, the manuscript deserves consideration. However, it cannot be published in the present form. Reasons for refusal and suggestions for improvement are given below:

• what was the population of Bwaise?

The population of Bwaise has now been clearly stated per zone in the manuscript.

• Forty-two participants were male caregivers (Table 1): however, gender is not considered in the analyses.

Thank you for pointing out this oversight. We have added gender as a covariate in our analyses and have updated the relevant sections of the manuscript.

• Active tobacco smoke as a risk factor for respiratory symptoms on its own merits is hardly considered. According to Table 1, in 41 of 284 households included in the survey (14.4%) there was a residing member who was an active smoker. Risks from environmental factors are estimated without any distinction between active and passive smokers.

Thank you for the comment. We agree that active tobacco smoke is an important risk factor for respiratory symptoms and have included this information in our revised manuscript. In this study, we assessed active smoking for adults.

• Similarly, data on active and passive tobacco smoke are unclear: lines 147-48, 151 and 171 describe respectively “smoking”, “household smoking” and “indoor smoking” as a confounder. Do these terms correspond each other? Do they include exposure to passive tobacco smoke?

We apologize for any confusion caused by our use of different terms to describe smoking and exposure to tobacco smoke. It was active smoking and we have harmonized this for consistency.

• Some details are missed in the description of the conduct of the survey. How many data collectors / interviewers? Which the number of persons refused to be interviewed or could to be interviewed for other reasons?

Thank you for your feedback on the survey conduct. We worked with 6 research assistants (indicated in the methods under section “Data collection and measurements” on page 5) and we had no refusals in this study.

• Throughout the text, I have not been able to find the absolute number of childlren which were included in the survey. I have only learnt that among the 284 households, 187 had at least a child.

We apologize for not providing the absolute number of children included in the survey. We have updated the manuscript to reflect that a total of 230 children were included in our study----page 9, line 229 and page 9 line 210-11.
• Tables 3-6 are too crowded (particularly tables 3 and 5) and difficult to read. Wouldn’t it be possible to produce a summary index for the 5 respiratory problems which are considered?

We understand that Tables 3 and 5 were difficult to read and have taken steps to improve them for clarity. We have now provided these tables as part of the supplementary files. We wanted to zoom in the specific respiratory problems than a summary index because there is not even a clear framework for generating summary index.

• Tables 3 and 5 report risk estimates based on “bivariate” (monovariate?) analyses whereas Tables 4 and 6 report results of multivariate analyses. Which criteria were used for selecting risk factors to be included in the latter tables?

Thank you for bringing this to our attention. We have added information on the criteria used for selecting risk factors in the multivariate analyses and have made this more explicit in the revised manuscript. We have now provided a DAG which guided the variables to consider for modelling.

Reviewer #2:

The manuscript entitled “Indoor air pollutants and respiratory symptoms among residents of an informal urban setting in Uganda: a cross-sectional study” describes the relationship between Indoor Air Pollution and respiratory symptoms in a slum area of the Uganda. The major strength of the manuscript is that it provides useful empirical data collected in difficult settings. The major limitation is its cross-sectional nature, the small sample size, and some modelling strategy.

Thank you for your detailed review of our manuscript. We appreciate your comments and suggestions and have addressed them as follows.

Introduction:

Overall, the introduction is too much focused on the relationship between Indoor Air Pollution (IAP) and Acute Respiratory Infection (ARI), while the aim of the study is to assess a more general the relationship between IAQ and respiratory health, using different set of non-specific respiratory symptoms.

**Response**: We have revised the introduction to provide a broader perspective of the relationship between indoor air pollutants and respiratory health, including other respiratory symptoms that may be linked to indoor air pollution. We have also included references to support these additional points.

Line 38 Page 1: I would expand the section briefly introducing also other respiratory health problems that might be linked to IAP as “irritation of the eyes, nose, and throat”, “lung development”, and “asthma”.

**Response**: Thank you for your suggestion to expand on the respiratory health problems linked to IAP. We have revised the paragraph to include additional respiratory health problems such as irritation of the eyes, nose, and throat, and asthma, as well as long-term health effects associated with exposure to indoor air pollutants such as pneumonia, chronic obstructive pulmonary disease, heart disease, and lung cancer. This additional information helps to provide a more comprehensive understanding of the impact of IAQ on respiratory health.

Page 2, Line 47: “Inadequate housing conditions in Ugandan informal settings can negatively affect IAQ and hence partly account for disparities in the burden of ARIs”. Please indicate a reference.

**Response**: We have added a reference to support the statement that inadequate housing conditions in Ugandan informal settings can negatively affect IAQ on page 2, line 49.

Methods:

Sampling strategy: Please provide the total number of inhabitants/households of the Bwaise slum. In addition, it should be provided the number of inhabitants/households for each district of Bwaise (Bwaise I, II, III).

**Response:** Sampling strategy: We have provided the total number of inhabitants/households of the Bwaise slum and the number of inhabitants/households for each district of Bwaise (Bwaise I, II, III) - see page 4, lines 121 to 125.

**Comment**: Data collection and measurements: Please report that indoor residual spraying was self-reported (in the current version of the manuscript it is not clear)

**Response:** Data collection and measurements: We have clarified that indoor residual spraying was self-reported in the revised manuscript (page 6, lines 136-137).


Statistical analysis:

Even if authors collect quantitatively the IAP and state in the introduction that: “ relying on sources of IAP may be a good proxy of exposure, objectively measured quantitative exposure assessments can be more helpful in assessing the health effects of exposure”, they eventually dichotomise the exposure, without testing possible linear or non linear effect on a continuous scale of the exposure. I would suggest modelling the relationship on a continuous scale, as well as testing linearity and non-linearity of the relationship between exposure and outcome.

Thank you for the suggestion. We have used a continuous exposure though we scaled it and a variable equal to 1/10^th^ of PM2.5 for easier interpretation i.e “ 10 unit increase in pm2.5 was associated with…..”. page 7, line 239. The linearity assumption was violated and this was addressed by scaling. This was log-transformed to achieve linearity.

Bivariate modelling seems correct; however, I do not agree with the multivariable modelling strategy. Authors adjust all models for age, gender and smoking recognizing them as a priori confounders.
Thank you for raising this concern. We have now modelled variables based on the simple Directed Acyclic Graph -DAG (the DAG is now provided as part of the supplementary file 3). We have also described the analysis approach on page 7, lines 240 - 244.

However, for each exposure-outcome model they decided to include as additional covariate all variables having a p-value <0.20. If would suggest to not adapt this data-driven selection of confounder, especially in a small dataset as the one of this study. I will opt for a-priori selection of a set of confounders. For instance, some analyses are not adjusted for Household income, even if I see it as a confounder in all exposure-outcome relationship. A re-run of analysis using a-priori selected confounders is essential for the manuscript to be considered for publication.

Thank you for the suggestion. We have now used theory driven approach based on the DAG. We have provided the DAG, statistical analysis section and actual analysis.

As an example, let’s take Table 6, Phlegm outcome. Here estimate for Cooking from outside the living room is adjusted for PM2.5 concentration. PM2.5 concentration is not a confounder, but rather a mediator.

Thank you for the concern raised. We believe that outdoor cooking could influence PM2.5 but is also associated with respiratory health on its own. We agree PM2.5 may be a mediator of the path between outdoor cooking and respiratory problem but our exposure is not outdoor cooking but PM.25 concentration and other indoor air pollutant sources. Outdoor is only a parent of the exposure and therefore we have to control it (treat it as confounder) as per these assumptions. Thank you.

Results:

I discourage the use of the p-value categorizing in 0.05,0.005,0.001. In all results table is not clear why some estimates are presented in bold font, and others not. It does not seem to be due to the statistical significance, because asterisk and bold font do not correspond. Please remove asterisk, significance test, and report only point estimates and confidence intervals.

We have removed the p-values and the asterisks and have reported only point estimates and confidence intervals.

Discussion:

**Comment**: Page 15 Line 239: How authors motivate the high proportion of respiratory symptoms in the target population? In the sample size computation, they assumed a 20% prevalence of respiratory symptoms, while in the study population they found proportion >80%.
**Response**: Thank you for this comment. We have acknowledged in our discussion that the prevalence of respiratory symptoms in our study population was much higher than the 20% prevalence we had assumed in our sample size calculation. We have also explained that the high prevalence of respiratory symptoms in the study population may be attributed to the high levels of IAP in the study area and the widespread use of solid fuels for cooking and heating as compared to the study which provided the p for sample size estimation. Additionally, we noted that the study by Siddharthan, Grigsby (23) was conducted in rural and urban Uganda, while our study was conducted in an informal settlement in Kampala. Informal settlements are known to have significantly poorer IAQ conditions, which may also explain the higher prevalence of respiratory symptoms observed in our study. Overall, the high prevalence of respiratory symptoms in the study population underscores the urgent need for interventions to improve indoor air quality in slum settings.

Page 16 Line 254: Authors state “This study found that the risk of shortness of breath was 44% lower among adults in households whose cooking place was outside their living house than those who cooked from inside their living spaces. […] Indeed, previous studies have reported a higher concentration of these pollutants in homes where cooking was done indoors (43).” Authors can check this relationship using their own data, since they have collected information on both cooking location and indoor air pollutants.

We have checked the relationship between cooking location and indoor air pollutants using our data and have included the results in the revised manuscript. Our data however did not show significant differences in the lower levels of indoor air pollutants between to those who cooked indoors and outdoors (page 15 line 549, and page 16, line 703-704).

Thank you so much,

Solomon Wafula (on behalf of all authors)
