# Supplementary material for: Indoor air pollutants and respiratory symptoms among residents of an informal urban settlement in Uganda: A cross-sectional study
Source: PLoS One. 2023 Aug 17;18(8):e0290170. doi: 10.1371/journal.pone.0290170 (PMC10434877; doi:10.1371/journal.pone.0290170)
Supplement: Peer review history

# Peer review: Decision Letter 0

**DOI:** 10.1371/journal.pone.0290170.r001

**Authors:** Francesco Barone-Adesi

23 Mar 2023

PONE-D-22-35748Indoor air pollutants and respiratory symptoms among residents of an informal urban setting in Uganda: a cross-sectional studyPLOS ONE

Dear Dr. Wafula,

Thank you for submitting your manuscript to PLOS ONE. After careful consideration, we feel that it has merit but does not fully meet PLOS ONE’s publication criteria as it currently stands. Therefore, we invite you to submit a revised version of the manuscript that addresses the points raised during the review process.

Statistical analysis needs to be revised. In particular:

1) an analysis using exposure on the continuous scale should be performed, or a valid reason not to do it should be provided (Reviewer 2)

2) the multivariable analysis should be conducted on an a-priori set of confounders, not basing on data-driven strategies (Reviewer 2).

3) the use of p-value categorization should be avoided (Reviewer 2)

Some additional information needs to be provided. In particular:

1) Number of inhabitants in the different Bwaise districts (Reviewer 1/ Reviewer 2)

2) proportion of non-respondents (Reviewer 1)

Please also consider these additional comments from my side:

In table 2, the line about main fuel type is not clear. I assume that about 95% of household used biomass. Please correct the table accordingly.

According to the methods section, all children under five years in the selected households were included in the analysis. However, in the results section is reported that analysis was carried out on 230 children out of 284 households, less than one per household. Could you please double-check this number?

In the results section, you reported that prevalence of wheezing was 2.26 higher among single participants compared to married ones. However, this is the result of the univariable analysis, which is not adjusted for age, gender, smoking, etc. if you want to analyze this association (even if it is out of the main scope of the paper), you should report the adjusted results.

Apparently, the paragraph titled “Associations between indoor air conditions and respiratory problems among parent-reported respiratory problems among children” include only one sentence. It should be merged with the following one.

Minor issues

At line 234 you may want to say “the effect of fuel type” instead of “The predictive value of fuel type”

Line 246 “…and the finding is consistent” please cancel “and”

Line 247 for sake of clarity, you may want to say “Indoor PM concentration is related to inflammation and a decrease in lung function”

Line 264 please use sensibilization instead of sensitization

Line 287 please refer to “Simoni and colleagues”

Please submit your revised manuscript by May 07 2023 11:59PM. If you will need more time than this to complete your revisions, please reply to this message or contact the journal office at plosone@plos.org. When you're ready to submit your revision, log on to https://www.editorialmanager.com/pone/ and select the 'Submissions Needing Revision' folder to locate your manuscript file.

Please include the following items when submitting your revised manuscript:

- A rebuttal letter that responds to each point raised by the academic editor and reviewer(s). You should upload this letter as a separate file labeled 'Response to Reviewers'.
- A marked-up copy of your manuscript that highlights changes made to the original version. You should upload this as a separate file labeled 'Revised Manuscript with Track Changes'.
- An unmarked version of your revised paper without tracked changes. You should upload this as a separate file labeled 'Manuscript'.

If you would like to make changes to your financial disclosure, please include your updated statement in your cover letter. Guidelines for resubmitting your figure files are available below the reviewer comments at the end of this letter.

If applicable, we recommend that you deposit your laboratory protocols in protocols.io to enhance the reproducibility of your results. Protocols.io assigns your protocol its own identifier (DOI) so that it can be cited independently in the future. For instructions see: https://journals.plos.org/plosone/s/submission-guidelines#loc-laboratory-protocols. Additionally, PLOS ONE offers an option for publishing peer-reviewed Lab Protocol articles, which describe protocols hosted on protocols.io. Read more information on sharing protocols at https://plos.org/protocols?utm_medium=editorial-email&utm_source=authorletters&utm_campaign=protocols.

We look forward to receiving your revised manuscript.

Kind regards,

Francesco Barone-Adesi

Academic Editor

PLOS ONE

Journal Requirements:

When submitting your revision, we need you to address these additional requirements.

1. Please ensure that your manuscript meets PLOS ONE's style requirements, including those for file naming. The PLOS ONE style templates can be found at

https://journals.plos.org/plosone/s/file?id=wjVg/PLOSOne_formatting_sample_main_body.pdf and

https://journals.plos.org/plosone/s/file?id=ba62/PLOSOne_formatting_sample_title_authors_affiliations.pdf

2. Please note that the supplementary data file "SI Data. Dataset for children information" contains potentially identifying information (i.e. names of the children). These should be anonymised as this information may breach patient confidentiality and these specific case details should not be included.

3. You indicated that you had ethical approval for your study. In your Methods section, please ensure you have also stated whether you obtained consent from parents or guardians of the minors (participants under the age of 18 years) included in the study or whether the research ethics committee or IRB specifically waived the need for their consent.

4. We note that Figure 1 in your submission contain map images which may be copyrighted. All PLOS content is published under the Creative Commons Attribution License (CC BY 4.0), which means that the manuscript, images, and Supporting Information files will be freely available online, and any third party is permitted to access, download, copy, distribute, and use these materials in any way, even commercially, with proper attribution. For these reasons, we cannot publish previously copyrighted maps or satellite images created using proprietary data, such as Google software (Google Maps, Street View, and Earth). For more information, see our copyright guidelines: http://journals.plos.org/plosone/s/licenses-and-copyright.

We require you to either (1) present written permission from the copyright holder to publish these figures specifically under the CC BY 4.0 license, or (2) remove the figures from your submission:

 a. You may seek permission from the original copyright holder of Figure 1 to publish the content specifically under the CC BY 4.0 license. 

We recommend that you contact the original copyright holder with the Content Permission Form (http://journals.plos.org/plosone/s/file?id=7c09/content-permission-form.pdf) and the following text:

“I request permission for the open-access journal PLOS ONE to publish XXX under the Creative Commons Attribution License (CCAL) CC BY 4.0 (http://creativecommons.org/licenses/by/4.0/). Please be aware that this license allows unrestricted use and distribution, even commercially, by third parties. Please reply and provide explicit written permission to publish XXX under a CC BY license and complete the attached form.”

Please upload the completed Content Permission Form or other proof of granted permissions as an "Other" file with your submission.

In the figure caption of the copyrighted figure, please include the following text: “Reprinted from [ref] under a CC BY license, with permission from [name of publisher], original copyright [original copyright year].”

 b. If you are unable to obtain permission from the original copyright holder to publish these figures under the CC BY 4.0 license or if the copyright holder’s requirements are incompatible with the CC BY 4.0 license, please either i) remove the figure or ii) supply a replacement figure that complies with the CC BY 4.0 license. Please check copyright information on all replacement figures and update the figure caption with source information. If applicable, please specify in the figure caption text when a figure is similar but not identical to the original image and is therefore for illustrative purposes only.

The following resources for replacing copyrighted map figures may be helpful:

USGS National Map Viewer (public domain): http://viewer.nationalmap.gov/viewer/

The Gateway to Astronaut Photography of Earth (public domain): http://eol.jsc.nasa.gov/sseop/clickmap/

Maps at the CIA (public domain): https://www.cia.gov/library/publications/the-world-factbook/index.html and https://www.cia.gov/library/publications/cia-maps-publications/index.html

NASA Earth Observatory (public domain): http://earthobservatory.nasa.gov/

Landsat: http://landsat.visibleearth.nasa.gov/

USGS EROS (Earth Resources Observatory and Science (EROS) Center) (public domain): http://eros.usgs.gov/#

Natural Earth (public domain): http://www.naturalearthdata.com/

5. We note you have included a table to which you do not refer in the text of your manuscript. Please ensure that you refer to Table 3 in your text; if accepted, production will need this reference to link the reader to the Table.

[Note: HTML markup is below. Please do not edit.]

Reviewers' comments:

Reviewer's Responses to Questions

**Comments to the Author**

1. Is the manuscript technically sound, and do the data support the conclusions?

The manuscript must describe a technically sound piece of scientific research with data that supports the conclusions. Experiments must have been conducted rigorously, with appropriate controls, replication, and sample sizes. The conclusions must be drawn appropriately based on the data presented. 

Reviewer #1: Partly

Reviewer #2: Partly

**********

2. Has the statistical analysis been performed appropriately and rigorously? 

Reviewer #1: Yes

Reviewer #2: No

**********

3. Have the authors made all data underlying the findings in their manuscript fully available?

The PLOS Data policy requires authors to make all data underlying the findings described in their manuscript fully available without restriction, with rare exception (please refer to the Data Availability Statement in the manuscript PDF file). The data should be provided as part of the manuscript or its supporting information, or deposited to a public repository. For example, in addition to summary statistics, the data points behind means, medians and variance measures should be available. If there are restrictions on publicly sharing data—e.g. participant privacy or use of data from a third party—those must be specified.

Reviewer #1: Yes

Reviewer #2: Yes

**********

4. Is the manuscript presented in an intelligible fashion and written in standard English?

PLOS ONE does not copyedit accepted manuscripts, so the language in submitted articles must be clear, correct, and unambiguous. Any typographical or grammatical errors should be corrected at revision, so please note any specific errors here.

Reviewer #1: Yes

Reviewer #2: Yes

**********

5. Review Comments to the Author

Please use the space provided to explain your answers to the questions above. You may also include additional comments for the author, including concerns about dual publication, research ethics, or publication ethics. (Please upload your review as an attachment if it exceeds 20,000 characters)

Reviewer #1: This paper is one of the first aimed at providing some information on the effects of environmental pollution on the health of residents in Uganda. Some findings (eg the protective effect of outdoor cooking) are convincing and may have an impact on public health policies.

The authors honestly recognize the limitations of their study.

Thus, the manuscript deserves consideration. However, it cannot be published in the present form. Reasons for refusal and suggestions for improvement are given below:

• what was the population of Bwaise?

• Forty-two participants were male caregivers (Table 1): however, gender is not considered in the analyses.

• Active tobacco smoke as a risk factor for respiratory symptoms on its own merits is hardly considered. According to Table 1, in 41 of 284 households included in the survey (14.4%) there was a residing member who was an active smoker. Risks from environmental factors are estimated without any distinction between active and passive smokers.

• Similarly, data on active and passive tobacco smoke are unclear: lines 147-48, 151 and 171 describe respectively “smoking”, “household smoking” and “indoor smoking” as a confounder. Do these terms correspond each other? Do they include exposure to passive tobacco smoke?

• Some details are missed in the description of the conduct of the survey How many data collectors / interviewers? Which the number of persons refused to be interviewed or could to be interviewed for other reasonss?

• Throughout the text, I have not been able to find the absolute number of childlren which were included in the survey. I have only learnt that among the 284 households, 187 had at least a child.

• Tables 3-6 are too crowded (particularly tables 3 and 5) and difficult to read. Wouldn’t it be possible to produce a summary index for the 5 respiratory problems which are considered?

• Tables 3 and 5 report risk estimates based on “bivariate” (monovariate?) analyses whereas Tables 4 and 6 report results of multivariate analyses. Which criteria were used for selecting risk factors to be included in the latter tables?

Reviewer #2: The manuscript entitled “Indoor air pollutants and respiratory symptoms among residents of an informal urban setting in Uganda: a cross-sectional study” describes the relationship between Indoor Air Pollution and respiratory symptoms in a slum area of the Uganda. The major strength of the manuscript is that it provides useful empirical data collected in difficult settings. The major limitation is its cross-sectional nature, the small sample size, and some modelling strategy.

Introduction:

Overall, the introduction is too much focused on the relationship between Indoor Air Pollution (IAP) and Acute Respiratory Infection (ARI), while the aim of the study is to assess a more general the relationship between IAQ and respiratory health, using different set of non-specific respiratory symptoms.

Line 38 Page 1: I would expand the section briefly introducing also other respiratory health problems that might be linked to IAP as “irritation of the eyes, nose, and throat”, “lung development”, and “asthma”.

Page 2, Line 47: “Inadequate housing conditions in Ugandan informal settings can negatively affect IAQ and hence partly account for disparities in the burden of ARIs”. Please indicate a reference.

Methods:

Sampling strategy: Please provide the total number of inhabitants/households of the Bwaise slum. In addition, it should be provided the number of inhabitants/households for each district of Bwaise (Bwaise I, II, III).

Data collection and measurements: Please report that indoor residual spraying was self-reported (in the current version of the manuscript it is not clear)

Statistical analysis:

Even if authors collect quantitatively the IAP and state in the introduction that: “ relying on sources of IAP may be a good proxy of exposure, objectively measured quantitative exposure assessments can be more helpful in assessing the health effects of exposure”, they eventually dichotomise the exposure, without testing possible linear or non linear effect on a continuous scale of the exposure. I would suggest modelling the relationship on a continuous scale, as well as testing linearity and non-linearity of the relationship between exposure and outcome.

Bivariate modelling seems correct, however I do not agree with the multivariable modelling strategy. Authors adjust all models for age, gender and smoking recognizing them as a priori confounders.

However, for each exposure-outcome model they decided to include as additional covariate all variables having a p-value <0.20. If would suggest to not adapt this data-driven selection of confounder, especially in a small dataset as the one of this study. I will opt for a-priori selection of a set of confounders. For instance some analyses are not adjusted for Household income, even if I see it as a confounder in all exposure-outcome relationship. A re-run of analysis using a-priori selected confounders is essential for the manuscript to be considered for publication.

As an example let’s take Table 6, Phlegm outcome. Here estimate for Cooking from outside the living room is adjusted for PM2.5 concentration. PM2.5 concentration is not a confounder, but rather a mediator.

Results:

I discourage the use of the p-value categorizing in 0.05,0.005,0.001. In all results table is not clear why some estimates are presented in bold font, and others not. It does not seem to be due to the statistical significance, because asterisk and bold font do not correspond. Please remove asterisk, significance test, and report only point estimates and confidence intervals.

Discussion:

Page 15 Line 239: How authors motivate the high proportion of respiratory symptoms in the target population? In the sample size computation, they assumed a 20% prevalence of respiratory symptoms, while in the study population they found proportion >80%.

Page 16 Line 254: Authors state “This study found that the risk of shortness of breath was 44% lower among adults in households whose cooking place was outside their living house than those who cooked from inside their living spaces. […] Indeed, previous studies have reported a higher concentration of these pollutants in homes where cooking was done indoors (43).”

Authors can check this relationship using their own data, since they have collected information on both cooking location and indoor air pollutants.

**********

6. PLOS authors have the option to publish the peer review history of their article (what does this mean?). If published, this will include your full peer review and any attached files.

If you choose “no”, your identity will remain anonymous but your review may still be made public.

**Do you want your identity to be public for this peer review?** For information about this choice, including consent withdrawal, please see our Privacy Policy.

Reviewer #1: No

Reviewer #2: **Yes: **Giovenale Moirano

**********

[NOTE: If reviewer comments were submitted as an attachment file, they will be attached to this email and accessible via the submission site. Please log into your account, locate the manuscript record, and check for the action link "View Attachments". If this link does not appear, there are no attachment files.]

While revising your submission, please upload your figure files to the Preflight Analysis and Conversion Engine (PACE) digital diagnostic tool, https://pacev2.apexcovantage.com/. PACE helps ensure that figures meet PLOS requirements. To use PACE, you must first register as a user. Registration is free. Then, login and navigate to the UPLOAD tab, where you will find detailed instructions on how to use the tool. If you encounter any issues or have any questions when using PACE, please email PLOS at figures@plos.org. Please note that Supporting Information files do not need this step.

# Author comment: Author response to Decision Letter 0

**DOI:** 10.1371/journal.pone.0290170.r002

9 Jun 2023

Makerere University

School of Public Health

P.O Box 7072, Kampala

27th May 2023

The Editor,

PLOS ONE Journal. 

Re: Submission of a revised review article for publication 

We would like to express our gratitude to you for providing us with the opportunity to revise our manuscript titled “Indoor air pollutants and respiratory symptoms among residents of an informal urban settlement in Uganda: a cross-sectional study”. We are also thankful to the peer reviewers for their valuable insights, which have significantly enhanced the quality of our work.

In response to the reviewers' comments, we have made diligent efforts to address each point raised, striving to incorporate their suggestions and improve the manuscript accordingly. We are now pleased to submit the revised version of our manuscript for your consideration. 

Sincerely, 

Solomon Wafula

 

#General comments 

Statistical analysis needs to be revised. In particular:

1) an analysis using exposure on the continuous scale should be performed, or a valid reason not to do it should be provided (Reviewer 2)

We have now performed re-analysis using exposure on the continuous scale where necessary in the revised manuscript. A detailed response is under specific comments. 

2) the multivariable analysis should be conducted on an a-priori set of confounders, not basing on data-driven strategies (Reviewer 2).

Thank you for the suggestion. We have conducted the multivariable analysis on an a-priori set of confounders based on the DAG theory and we provide rationale for the choice of priori set of confounders in the methods. 

3) the use of p-value categorization should be avoided (Reviewer 2)

Response: We have avoided the use of p-value categorization in the revised manuscript as per your suggestion. 

Some additional information needs to be provided. In particular:

1) Number of inhabitants in the different Bwaise districts (Reviewer 1/ Reviewer 2)

We have provided the number of inhabitants in the different Bwaise zones…. on page 4

2) proportion of non-respondents (Reviewer 1)

Thank you for the suggestion but in this study, we did not have any non-response. 

Please also consider these additional comments from my side:

In table 2, the line about main fuel type is not clear. I assume that about 95% of household used biomass. Please correct the table accordingly.

We have corrected table 2 to make the line about main fuel type clearer.

According to the methods section, all children under five years in the selected households were included in the analysis. However, in the results section is reported that analysis was carried out on 230 children out of 284 households, less than one per household. Could you please double-check this number?

Thank you for the comment. We understand that the number of children included in the analysis is lower than expected but we confirm that this number is correct. We found fewer children (n = 230) than households (n = 284) as many households had no children. Having a child under 5 was not a requirement for a household to be included in the study. 

In the results section, you reported that prevalence of wheezing was 2.26 higher among single participants compared to married ones. However, this is the result of the univariable analysis, which is not adjusted for age, gender, smoking, etc. if you want to analyze this association (even if it is out of the main scope of the paper), you should report the adjusted results.

Thank you for your observation. We have now reported the updated adjusted results. However, since the study focused on indoor air conditions and respiratory problem, we do not provide narratives for the association between socio-demographic variables and respiratory problems. 

Apparently, the paragraph titled “Associations between indoor air conditions and respiratory problems among parent-reported respiratory problems among children” include only one sentence. It should be merged with the following one.

We have merged the two paragraphs combining the narratives from bivariate analysis and multivariable analysis

Minor issues

Comment: At line 234 you may want to say “the effect of fuel type” instead of “The predictive value of fuel type”

Response: We have corrected the wording to read “the effect of fuel type” instead of “The predictive value of fuel type.”

Comment: Line 246 “…and the finding is consistent” please cancel “and”

Response: We have removed “and” from line 246 for clarity.

Comment: Line 247 for sake of clarity, you may want to say “Indoor PM concentration is related to inflammation and a decrease in lung function”

Response: We have revised the wording at line 247 to read “Indoor PM concentration is related to inflammation and decreased lung function” for clarity.

Comment: Line 264 please use sensibilization instead of sensitization

Response: We have used “sensibilization” instead of “sensitization” in line 264.

Comment: Line 287 please refer to “Simoni and colleagues”

Response: We have referred to “Simoni et al.” at line 287

Reviewer #1: This paper is one of the first aimed at providing some information on the effects of environmental pollution on the health of residents in Uganda. Some findings (eg the protective effect of outdoor cooking) are convincing and may have an impact on public health policies.

The authors honestly recognize the limitations of their study.

Response: Thank you for reviewing our manuscript and providing constructive feedback. We appreciate the time and effort you have taken to read and evaluate our study. Below is our point-by-point response to your comments:

Thus, the manuscript deserves consideration. However, it cannot be published in the present form. Reasons for refusal and suggestions for improvement are given below:

• what was the population of Bwaise?

The population of Bwaise has now been clearly stated per zone in the manuscript. 

• Forty-two participants were male caregivers (Table 1): however, gender is not considered in the analyses.

Thank you for pointing out this oversight. We have added gender as a covariate in our analyses and have updated the relevant sections of the manuscript.

• Active tobacco smoke as a risk factor for respiratory symptoms on its own merits is hardly considered. According to Table 1, in 41 of 284 households included in the survey (14.4%) there was a residing member who was an active smoker. Risks from environmental factors are estimated without any distinction between active and passive smokers.

Thank you for the comment. We agree that active tobacco smoke is an important risk factor for respiratory symptoms and have included this information in our revised manuscript. In this study, we assessed active smoking for adults. 

• Similarly, data on active and passive tobacco smoke are unclear: lines 147-48, 151 and 171 describe respectively “smoking”, “household smoking” and “indoor smoking” as a confounder. Do these terms correspond each other? Do they include exposure to passive tobacco smoke?

We apologize for any confusion caused by our use of different terms to describe smoking and exposure to tobacco smoke. It was active smoking and we have harmonized this for consistency. 

• Some details are missed in the description of the conduct of the survey. How many data collectors / interviewers? Which the number of persons refused to be interviewed or could to be interviewed for other reasons?

Thank you for your feedback on the survey conduct. We worked with 6 research assistants (indicated in the methods under section “Data collection and measurements” on page 5) and we had no refusals in this study.

• Throughout the text, I have not been able to find the absolute number of childlren which were included in the survey. I have only learnt that among the 284 households, 187 had at least a child.

We apologize for not providing the absolute number of children included in the survey. We have updated the manuscript to reflect that a total of 230 children were included in our study----page 9, line 229 and page 9 line 210-11.

• Tables 3-6 are too crowded (particularly tables 3 and 5) and difficult to read. Wouldn’t it be possible to produce a summary index for the 5 respiratory problems which are considered?

We understand that Tables 3 and 5 were difficult to read and have taken steps to improve them for clarity. We have now provided these tables as part of the supplementary files. We wanted to zoom in the specific respiratory problems than a summary index because there is not even a clear framework for generating summary index. 

• Tables 3 and 5 report risk estimates based on “bivariate” (monovariate?) analyses whereas Tables 4 and 6 report results of multivariate analyses. Which criteria were used for selecting risk factors to be included in the latter tables?

Thank you for bringing this to our attention. We have added information on the criteria used for selecting risk factors in the multivariate analyses and have made this more explicit in the revised manuscript. We have now provided a DAG which guided the variables to consider for modelling. 

Reviewer #2: 

The manuscript entitled “Indoor air pollutants and respiratory symptoms among residents of an informal urban setting in Uganda: a cross-sectional study” describes the relationship between Indoor Air Pollution and respiratory symptoms in a slum area of the Uganda. The major strength of the manuscript is that it provides useful empirical data collected in difficult settings. The major limitation is its cross-sectional nature, the small sample size, and some modelling strategy.

Thank you for your detailed review of our manuscript. We appreciate your comments and suggestions and have addressed them as follows.

Introduction:

Overall, the introduction is too much focused on the relationship between Indoor Air Pollution (IAP) and Acute Respiratory Infection (ARI), while the aim of the study is to assess a more general the relationship between IAQ and respiratory health, using different set of non-specific respiratory symptoms.

Response: We have revised the introduction to provide a broader perspective of the relationship between indoor air pollutants and respiratory health, including other respiratory symptoms that may be linked to indoor air pollution. We have also included references to support these additional points.

Line 38 Page 1: I would expand the section briefly introducing also other respiratory health problems that might be linked to IAP as “irritation of the eyes, nose, and throat”, “lung development”, and “asthma”.

Response: Thank you for your suggestion to expand on the respiratory health problems linked to IAP. We have revised the paragraph to include additional respiratory health problems such as irritation of the eyes, nose, and throat, and asthma, as well as long-term health effects associated with exposure to indoor air pollutants such as pneumonia, chronic obstructive pulmonary disease, heart disease, and lung cancer. This additional information helps to provide a more comprehensive understanding of the impact of IAQ on respiratory health. 

Page 2, Line 47: “Inadequate housing conditions in Ugandan informal settings can negatively affect IAQ and hence partly account for disparities in the burden of ARIs”. Please indicate a reference.

Response: We have added a reference to support the statement that inadequate housing conditions in Ugandan informal settings can negatively affect IAQ on page 2, line 49.

Methods:

Sampling strategy: Please provide the total number of inhabitants/households of the Bwaise slum. In addition, it should be provided the number of inhabitants/households for each district of Bwaise (Bwaise I, II, III).

Response: Sampling strategy: We have provided the total number of inhabitants/households of the Bwaise slum and the number of inhabitants/households for each district of Bwaise (Bwaise I, II, III) - see page 4, lines 121 to 125.

Comment: Data collection and measurements: Please report that indoor residual spraying was self-reported (in the current version of the manuscript it is not clear)

Response: Data collection and measurements: We have clarified that indoor residual spraying was self-reported in the revised manuscript (page 6, lines 136-137).

Statistical analysis:

Even if authors collect quantitatively the IAP and state in the introduction that: “ relying on sources of IAP may be a good proxy of exposure, objectively measured quantitative exposure assessments can be more helpful in assessing the health effects of exposure”, they eventually dichotomise the exposure, without testing possible linear or non linear effect on a continuous scale of the exposure. I would suggest modelling the relationship on a continuous scale, as well as testing linearity and non-linearity of the relationship between exposure and outcome.

Thank you for the suggestion. We have used a continuous exposure though we scaled it and a variable equal to 1/10th of PM2.5 for easier interpretation i.e “ 10 unit increase in pm2.5 was associated with…..”. page 7, line 239. The linearity assumption was violated and this was addressed by scaling. This was log-transformed to achieve linearity. 

Bivariate modelling seems correct; however, I do not agree with the multivariable modelling strategy. Authors adjust all models for age, gender and smoking recognizing them as a priori confounders.

Thank you for raising this concern. We have now modelled variables based on the simple Directed Acyclic Graph -DAG (the DAG is now provided as part of the supplementary file 3). We have also described the analysis approach on page 7, lines 240 - 244.

However, for each exposure-outcome model they decided to include as additional covariate all variables having a p-value <0.20. If would suggest to not adapt this data-driven selection of confounder, especially in a small dataset as the one of this study. I will opt for a-priori selection of a set of confounders. For instance, some analyses are not adjusted for Household income, even if I see it as a confounder in all exposure-outcome relationship. A re-run of analysis using a-priori selected confounders is essential for the manuscript to be considered for publication.

Thank you for the suggestion. We have now used theory driven approach based on the DAG. We have provided the DAG, statistical analysis section and actual analysis. 

As an example, let’s take Table 6, Phlegm outcome. Here estimate for Cooking from outside the living room is adjusted for PM2.5 concentration. PM2.5 concentration is not a confounder, but rather a mediator.

Thank you for the concern raised. We believe that outdoor cooking could influence PM2.5 but is also associated with respiratory health on its own. We agree PM2.5 may be a mediator of the path between outdoor cooking and respiratory problem but our exposure is not outdoor cooking but PM.25 concentration and other indoor air pollutant sources. Outdoor is only a parent of the exposure and therefore we have to control it (treat it as confounder) as per these assumptions. Thank you. 

Results:

I discourage the use of the p-value categorizing in 0.05,0.005,0.001. In all results table is not clear why some estimates are presented in bold font, and others not. It does not seem to be due to the statistical significance, because asterisk and bold font do not correspond. Please remove asterisk, significance test, and report only point estimates and confidence intervals.

We have removed the p-values and the asterisks and have reported only point estimates and confidence intervals.

Discussion:

Comment: Page 15 Line 239: How authors motivate the high proportion of respiratory symptoms in the target population? In the sample size computation, they assumed a 20% prevalence of respiratory symptoms, while in the study population they found proportion >80%.

Response: Thank you for this comment. We have acknowledged in our discussion that the prevalence of respiratory symptoms in our study population was much higher than the 20% prevalence we had assumed in our sample size calculation. We have also explained that the high prevalence of respiratory symptoms in the study population may be attributed to the high levels of IAP in the study area and the widespread use of solid fuels for cooking and heating as compared to the study which provided the p for sample size estimation. Additionally, we noted that the study by Siddharthan, Grigsby (23) was conducted in rural and urban Uganda, while our study was conducted in an informal settlement in Kampala. Informal settlements are known to have significantly poorer IAQ conditions, which may also explain the higher prevalence of respiratory symptoms observed in our study. Overall, the high prevalence of respiratory symptoms in the study population underscores the urgent need for interventions to improve indoor air quality in slum settings.

Page 16 Line 254: Authors state “This study found that the risk of shortness of breath was 44% lower among adults in households whose cooking place was outside their living house than those who cooked from inside their living spaces. […] Indeed, previous studies have reported a higher concentration of these pollutants in homes where cooking was done indoors (43).” Authors can check this relationship using their own data, since they have collected information on both cooking location and indoor air pollutants.

We have checked the relationship between cooking location and indoor air pollutants using our data and have included the results in the revised manuscript. Our data however did not show significant differences in the lower levels of indoor air pollutants between to those who cooked indoors and outdoors (page 15 line 549, and page 16, line 703-704).

Thank you so much, 

Solomon Wafula (on behalf of all authors)

Attachment Submitted filename: Response to IAQ comments_27052023.docx Click here for additional data file.

# Peer review: Decision Letter 1

**DOI:** 10.1371/journal.pone.0290170.r003

**Authors:** Francesco Barone-Adesi

21 Jul 2023

PONE-D-22-35748R1Indoor air pollutants and respiratory symptoms among residents of an informal urban setting in Uganda: a cross-sectional studyPLOS ONE

Dear Dr. Wafula,

Thank you for submitting your manuscript to PLOS ONE. After careful consideration, we feel that it has merit but does not fully meet PLOS ONE’s publication criteria as it currently stands. Therefore, we invite you to submit a revised version of the manuscript that addresses the points raised during the review process.

Please submit your revised manuscript by Sep 04 2023 11:59PM. If you will need more time than this to complete your revisions, please reply to this message or contact the journal office at plosone@plos.org. When you're ready to submit your revision, log on to https://www.editorialmanager.com/pone/ and select the 'Submissions Needing Revision' folder to locate your manuscript file.

Please include the following items when submitting your revised manuscript:

- A rebuttal letter that responds to each point raised by the academic editor and reviewer(s). You should upload this letter as a separate file labeled 'Response to Reviewers'.
- A marked-up copy of your manuscript that highlights changes made to the original version. You should upload this as a separate file labeled 'Revised Manuscript with Track Changes'.
- An unmarked version of your revised paper without tracked changes. You should upload this as a separate file labeled 'Manuscript'.

If you would like to make changes to your financial disclosure, please include your updated statement in your cover letter. Guidelines for resubmitting your figure files are available below the reviewer comments at the end of this letter.

If applicable, we recommend that you deposit your laboratory protocols in protocols.io to enhance the reproducibility of your results. Protocols.io assigns your protocol its own identifier (DOI) so that it can be cited independently in the future. For instructions see: https://journals.plos.org/plosone/s/submission-guidelines#loc-laboratory-protocols. Additionally, PLOS ONE offers an option for publishing peer-reviewed Lab Protocol articles, which describe protocols hosted on protocols.io. Read more information on sharing protocols at https://plos.org/protocols?utm_medium=editorial-email&utm_source=authorletters&utm_campaign=protocols.

We look forward to receiving your revised manuscript.

Kind regards,

Francesco Barone-Adesi

Academic Editor

PLOS ONE

Journal Requirements:

Please review your reference list to ensure that it is complete and correct. If you have cited papers that have been retracted, please include the rationale for doing so in the manuscript text, or remove these references and replace them with relevant current references. Any changes to the reference list should be mentioned in the rebuttal letter that accompanies your revised manuscript. If you need to cite a retracted article, indicate the article’s retracted status in the References list and also include a citation and full reference for the retraction notice.

**Additional Editor Comments:**

SUMMARY OF COMMENTS

Most of the previous comments have been addressed. Some minor inconsistencies still need to be double-checked.

Required changes:

1) Lines 247 and Table 4: check data about PR for dampness and phlegm: 1.42 (line 250) or 16.5 (Table 4)? If PR = 16.5, add absolute number of observations.

2) Reader does not immediately appreciate that in both tables 3 and 4 prevalence rates for exposure to PM2.5 refer to 10 unit increase in concentration: a footnote would help.

3) Lines 345-347: where in the text or in the tables does the study reveal any differences between married and unmarried responders?

4) Make sure all acronyms are spelled out (e.g, IAQ: inner air quality?)

5) Authors should state for which additional covariates the model are adjusted in the manuscript or in the footnote of results table. Currently, they are only reported in the supplementary materials S3.

6) In table 2 please report that, for what concerns Main fuel type, Cooking from outside, Rearing pets, Carpets in living room and Home dampness (mould), the reported summary statistics represent percentage and absolute numbers.

Recommended changes:

1) In the analysis of PM2.5 and PM10 instead of reporting an increase of 10th, authors could report the effect for an increase of 20? Estimates for 10 unit increase seems slightly small.

[Note: HTML markup is below. Please do not edit.]

Reviewers' comments:

Reviewer's Responses to Questions

**Comments to the Author**

1. If the authors have adequately addressed your comments raised in a previous round of review and you feel that this manuscript is now acceptable for publication, you may indicate that here to bypass the “Comments to the Author” section, enter your conflict of interest statement in the “Confidential to Editor” section, and submit your "Accept" recommendation.

Reviewer #1: All comments have been addressed

Reviewer #2: (No Response)

********** 

2. Is the manuscript technically sound, and do the data support the conclusions?

The manuscript must describe a technically sound piece of scientific research with data that supports the conclusions. Experiments must have been conducted rigorously, with appropriate controls, replication, and sample sizes. The conclusions must be drawn appropriately based on the data presented. 

Reviewer #1: Yes

Reviewer #2: Yes

********** 

3. Has the statistical analysis been performed appropriately and rigorously? 

Reviewer #1: N/A

Reviewer #2: Yes

********** 

4. Have the authors made all data underlying the findings in their manuscript fully available?

The PLOS Data policy requires authors to make all data underlying the findings described in their manuscript fully available without restriction, with rare exception (please refer to the Data Availability Statement in the manuscript PDF file). The data should be provided as part of the manuscript or its supporting information, or deposited to a public repository. For example, in addition to summary statistics, the data points behind means, medians and variance measures should be available. If there are restrictions on publicly sharing data—e.g. participant privacy or use of data from a third party—those must be specified.

Reviewer #1: Yes

Reviewer #2: Yes

********** 

5. Is the manuscript presented in an intelligible fashion and written in standard English?

PLOS ONE does not copyedit accepted manuscripts, so the language in submitted articles must be clear, correct, and unambiguous. Any typographical or grammatical errors should be corrected at revision, so please note any specific errors here.

Reviewer #1: Yes

Reviewer #2: Yes

********** 

6. Review Comments to the Author

Please use the space provided to explain your answers to the questions above. You may also include additional comments for the author, including concerns about dual publication, research ethics, or publication ethics. (Please upload your review as an attachment if it exceeds 20,000 characters)

Reviewer #1: Current manuscript is much improved over the previous one and can be published. A final re-reading by the authors hunting for minor inconsistencies is needed.

Some minor points require rewriting, such as:

Lines 247 and Table 4: check data about PR for dampness and phlegm: 1.42 (line 250) or 16.5 (Table 4)????. If PR = 16.5, add absolute number of observations.

Reader does not immediately appreciate that in both tables 3 and 4 prevalence rates for exposure to PM2,5 refer to 10 unit increase in concentration: a footnote would help.

Lines 345-347: where in the text or in the tables does the study reveal any differences between married and unmarried respondere?

Make sure all acronyms are decoded (IAQ: inner air quality?)

Reviewer #2: The majority of my comments have been addresses.

Here I report only some additional comments.

1. In the analysis of PM2.5 and PM10 instead of reporting an increase of 10th, authors could report the effect for an increase of 20? Estimates for 10 unit increase seems slighlty small.

2. Authors should state for which additional covariates the model are adjusted in the manuscript or in the footnote of results table. At the moment are only reported in the supplementary materials S3.

3. In table 2 please report that for Main fuel type, Cooking from outside, Rearing pets ,Carpets in living room and

Home dampness (mould) the summary statistics represent percentage and absolute numbers.

********** 

7. PLOS authors have the option to publish the peer review history of their article (what does this mean?). If published, this will include your full peer review and any attached files.

If you choose “no”, your identity will remain anonymous but your review may still be made public.

**Do you want your identity to be public for this peer review?** For information about this choice, including consent withdrawal, please see our Privacy Policy.

Reviewer #1: No

Reviewer #2: **Yes: **Giovenale Moirano

**********

[NOTE: If reviewer comments were submitted as an attachment file, they will be attached to this email and accessible via the submission site. Please log into your account, locate the manuscript record, and check for the action link "View Attachments". If this link does not appear, there are no attachment files.]

While revising your submission, please upload your figure files to the Preflight Analysis and Conversion Engine (PACE) digital diagnostic tool, https://pacev2.apexcovantage.com/. PACE helps ensure that figures meet PLOS requirements. To use PACE, you must first register as a user. Registration is free. Then, login and navigate to the UPLOAD tab, where you will find detailed instructions on how to use the tool. If you encounter any issues or have any questions when using PACE, please email PLOS at figures@plos.org. Please note that Supporting Information files do not need this step.

# Author comment: Author response to Decision Letter 1

**DOI:** 10.1371/journal.pone.0290170.r004

25 Jul 2023

Makerere University

School of Public Health

P.O Box 7072, Kampala

22nd July 2023

The Editor,

PLOS ONE Journal. 

Re: Submission of a revised review article for publication 

We would like to express our gratitude to you for providing us with the opportunity to revise our manuscript titled “Indoor air pollutants and respiratory symptoms among residents of an informal urban settlement in Uganda: a cross-sectional study”. We are also thankful to the peer reviewers for their valuable insights, which have significantly enhanced the quality of our work.

In response to the reviewers' comments, we have made diligent efforts to address each point raised, striving to incorporate their suggestions and improve the manuscript accordingly. We are now pleased to submit the revised version of our manuscript for your consideration. 

Sincerely, 

Solomon Wafula

 

Required changes:

1) Lines 247 and Table 4: check data about PR for dampness and phlegm: 1.42 (line 250) or 16.5 (Table 4)? If PR = 16.5, add absolute number of observations.

Thanks for this observation. We have corrected the PR. We have now provided row by column tables for distribution of outcomes by several covariables in the supplementary file. 

2) Reader does not immediately appreciate that in both tables 3 and 4 prevalence rates for exposure to PM2.5 refer to 10 unit increase in concentration: a footnote would help.

Thank you for the comment, we now provide the footnote for the readers. 

3) Lines 345-347: where in the text or in the tables does the study reveal any differences between married and unmarried responders?

Thanks for the observation. We have removed variable gender in adjusted models because we were not convinced it was a good confounder to adjust for. We have therefore removed the discussion point on gender and wheezing. 

4) Make sure all acronyms are spelled out (e.g, IAQ: inner air quality?)

We have now spelled out IAQ throughout the manuscript. We have spelled other acronyms the first time they are used. 

5) Authors should state for which additional covariates the model are adjusted in the manuscript or in the footnote of results table. Currently, they are only reported in the supplementary materials S3.

Thanks for the suggestion, we have added the variables adjusted for under the footnotes. 

6) In table 2 please report that, for what concerns Main fuel type, Cooking from outside, Rearing pets, Carpets in living room and Home dampness (mould), the reported summary statistics represent percentage and absolute numbers.

Thank you. We have modified and provided footnote with this guidance. 

Recommended changes:

1) In the analysis of PM2.5 and PM10 instead of reporting an increase of 10th, authors could report the effect for an increase of 20? Estimates for 10 unit increase seems slightly small.

Thanks for the information, after performing a logarithmic transformation, we proceeded with using a 10-unit increase. The results remain informative and valuable for our analysis.

# Editor report: Decision Letter 2

**DOI:** 10.1371/journal.pone.0290170.r005

**Authors:** Francesco Barone-Adesi

3 Aug 2023

Indoor air pollutants and respiratory symptoms among residents of an informal urban settlement in Uganda: a cross-sectional study

PONE-D-22-35748R2

Dear Dr. Wafula,

We’re pleased to inform you that your manuscript has been judged scientifically suitable for publication and will be formally accepted for publication once it meets all outstanding technical requirements.

Within one week, you’ll receive an e-mail detailing the required amendments. When these have been addressed, you’ll receive a formal acceptance letter and your manuscript will be scheduled for publication.

An invoice for payment will follow shortly after the formal acceptance. To ensure an efficient process, please log into Editorial Manager at http://www.editorialmanager.com/pone/, click the 'Update My Information' link at the top of the page, and double check that your user information is up-to-date. If you have any billing related questions, please contact our Author Billing department directly at authorbilling@plos.org.

If your institution or institutions have a press office, please notify them about your upcoming paper to help maximize its impact. If they’ll be preparing press materials, please inform our press team as soon as possible -- no later than 48 hours after receiving the formal acceptance. Your manuscript will remain under strict press embargo until 2 pm Eastern Time on the date of publication. For more information, please contact onepress@plos.org.

Kind regards,

Francesco Barone-Adesi

Academic Editor

PLOS ONE

Additional Editor Comments:

Line 250 I think there is a bracket missing at the end of the sentence "((PR=1.31, 95% CI = 1.11 – 1.55 (Table 3)."

Line 256, I would suggest to use the same number of digits for the results reported in the text and in table 4 (i.e., PR= 13.9, 95% CI 3.16 – 60.9)

# Editor report: Acceptance letter

**DOI:** 10.1371/journal.pone.0290170.r006

**Authors:** Francesco Barone-Adesi

9 Aug 2023

PONE-D-22-35748R2 

Indoor air pollutants and respiratory symptoms among residents of an informal urban settlement in Uganda: a cross-sectional study 

Dear Dr. Wafula:

I'm pleased to inform you that your manuscript has been deemed suitable for publication in PLOS ONE. Congratulations! Your manuscript is now with our production department. 

If your institution or institutions have a press office, please let them know about your upcoming paper now to help maximize its impact. If they'll be preparing press materials, please inform our press team within the next 48 hours. Your manuscript will remain under strict press embargo until 2 pm Eastern Time on the date of publication. For more information please contact onepress@plos.org.

If we can help with anything else, please email us at plosone@plos.org. 

Thank you for submitting your work to PLOS ONE and supporting open access. 

Kind regards, 

PLOS ONE Editorial Office Staff

on behalf of

Professor Francesco Barone-Adesi 

Academic Editor

PLOS ONE
